# Classification of bursting patterns: A tale of two ducks

**DOI:** 10.1371/journal.pcbi.1009752

**Published:** 2022-02-24

**Authors:** Mathieu Desroches, John Rinzel, Serafim Rodrigues

**Affiliations:** 1 MathNeuro Team, Inria Sophia Antipolis Méditerranée Research Centre, Sophia Antipolis, France; 2 MCEN Team, Basque Centre for Applied Mathematics (BCAM), Bilbao, Bizkaia, Spain; 3 Center for Neural Science, New York University, New York, New York, United States of America; 4 Courant Institute for Mathematical Sciences, New York University, New York, New York, United States of America; 5 Ikerbasque, The Basque Science Foundation, Bilbao, Bizkaia, Spain; Inria, FRANCE

## Abstract

Bursting is one of the fundamental rhythms that excitable cells can generate either in response to incoming stimuli or intrinsically. It has been a topic of intense research in computational biology for several decades. The classification of bursting oscillations in excitable systems has been the subject of active research since the early 1980s and is still ongoing. As a by-product, it establishes analytical and numerical foundations for studying complex temporal behaviors in multiple timescale models of cellular activity. In this review, we first present the seminal works of Rinzel and Izhikevich in classifying bursting patterns of excitable systems. We recall a complementary mathematical classification approach by Bertram and colleagues, and then by Golubitsky and colleagues, which, together with the Rinzel-Izhikevich proposals, provide the state-of-the-art foundations to these classifications. Beyond classical approaches, we review a recent bursting example that falls outside the previous classification systems. Generalizing this example leads us to propose an extended classification, which requires the analysis of both fast and slow subsystems of an underlying slow-fast model and allows the dissection of a larger class of bursters. Namely, we provide a general framework for bursting systems with both subthreshold and superthreshold oscillations. A new class of bursters with at least 2 slow variables is then added, which we denote *folded-node bursters*, to convey the idea that the bursts are initiated or annihilated via a *folded-node* singularity. Key to this mechanism are so-called *canard* or *duck* orbits, organizing the underpinning excitability structure. We describe the 2 main families of folded-node bursters, depending upon the phase (active/spiking or silent/nonspiking) of the bursting cycle during which folded-node dynamics occurs. We classify both families and give examples of minimal systems displaying these novel bursting patterns. Finally, we provide a biophysical example by reinterpreting a generic conductance-based *episodic burster* as a folded-node burster, showing that the associated framework can explain its subthreshold oscillations over a larger parameter region than the fast subsystem approach.

## Author summary

Bursting is ubiquitous in cellular excitable rhythms and comes in a plethora of patterns, both experimentally recorded and reproduced through models. As these different patterns may reflect different coding or information properties, it is therefore crucial to develop modeling frameworks that can both capture them and understand their characteristics. In this review, we propose a comprehensive account of the main bursting classification systems that have been developed over the past 40 years, together with recent developments allowing us to extend these classifications. Based upon bifurcation theory and heavily reliant on timescale separation, these schemes take full advantage of the fast subsystem analysis, obtained when slow variables are frozen and considered as bifurcation parameters. We complement this classical view by showing that nontrivial slow subsystem may also encode key informations important to classify bursting rhythms, due to the presence of so-called folded-node singularities. We provide minimal idealized models as well as one generic conductance-based example displaying bursting oscillations that require our extended classification in order to be fully characterized. We also highlight examples of biological data that could be suitably revisited with the lenses of this extended classifications and could lead to new models of complex cellular activity.

## Introduction

The fascination of experimentalists, physicists, and mathematicians toward spontaneous and complex oscillations dates back to the early 20th century, particularly through observations of electrochemical systems [1]. Indeed, how can seemingly “inert subcomponents” assemble into “life,” in what is currently understood (in biophysics) as open multiscale (far from equilibrium) systems with dissipative structures? Van der pol was among the first scientists to exhibit equations with multiple timescales and a dissipative structure, which display oscillations akin to those observed in electrochemical systems and that indeed could not be explained by previous mathematical theories [2–4].

Despite remarkable advances, it is only relatively recently (since the 1980s) that a deeper understanding of certain types of nonlinear multiscale complex oscillations was made possible due to the development of a coherent mathematical theory and classification system for so-called *bursting* oscillations [5]. These developments have shaped mathematical and computational neuroscience, enriched experimental neuroscience, and also advanced the understanding of various biological systems. To further stimulate this field, the present review first provides a comprehensive account of several seminal works [6–9] as well as recent developments including our work on multiscale systems [10,11]. Finally, it proposes an extended classification framework, which we envisage will guide future developments of analytical, numerical, and modeling work on multiscale biological systems.

To contain the complexity of multiscale systems’ characterization, we will focus on the dynamics emerging from the interplay between the explicit timescales of a system of slow-fast Ordinary Differential Equations (ODEs) as this is the main framework underpinning the main bursting classification systems. Thus, we will consider models where the timescale ratio between fast and slow variables is explicitly given by a small positive parameter *ε*. This framework can also be applied to systems where timescale separation is not explicit though revealed through simulations. Such systems naturally emerge in various biological processes, and, to showcase the proposed framework at the end of this review, we will apply it to a biophysical neuron model. While this focus on only timescale separation circumvents the wider unresolved mathematical barriers in generally describing multiscale systems across spatiotemporal scales (e.g., via partial differential equations), it will enable us to obtain a significantly deeper insight on emergent timescale-induced dynamics. This will later inform these other multiscale approaches. Moreover, despite the relative apparent simplicity of minimal slow-fast ODEs, the associated theory is still in development. More importantly, such multi-timescale systems have already enabled remarkable predictions of complex oscillations, hence their relevance in computational biology [12–14] and neuroscience [15–17], among many application areas.

Depending on the dimension of fast and slow components, multiple timescale systems can reproduce key experimentally observed multiscale oscillations, in particular in neural recordings: *action potentials* or *spiking* behavior [18], *bursting* [19], *mixed-mode oscillations* [20]. As the main system parameters (e.g., an applied current) vary, the type of solution can change very rapidly and a minute parameter change may lead to a change in solution type upon a firing threshold crossing. In cases where such multiscale excitable systems can be modeled by slow-fast equations, then these sudden “explosive” transitions associated with excitability threshold crossings are organized by special solutions called *canards* or *ducks* [13,21,22]. These descriptors are used interchangeably in the literature, and we shall employ both terms.

**Fig 1:**
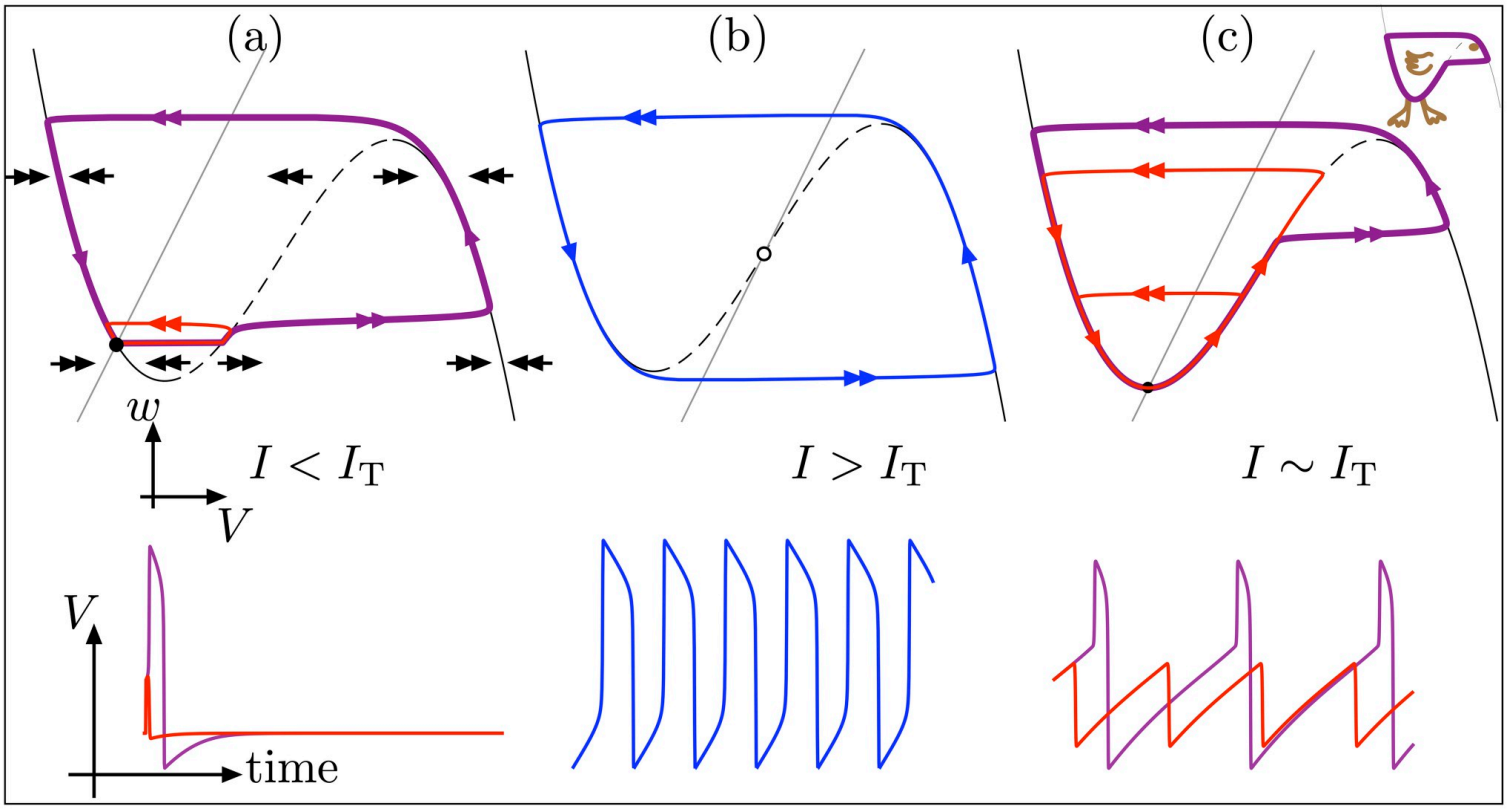
Ducks at the transition from rest to spikes in the FitzHugh–Nagumo model. The dynamics are represented in the phase plane (upper panels) and as illustrative time courses (lower panels). When increasing the applied current *I*, the system’s dynamics transitions from a stable equilibrium (rest state, black dot in (a)), which is excitable, to a strongly attracting limit cycle (repetitive spiking state, in blue in (b)). The excitable structure in panel (a) is further illustrated with 2 trajectories whose initial conditions are at the stable equilibrium (rest state) and with a step current of slightly different amplitude: One trajectory (in red) stays below threshold, while the other one (in purple) crosses threshold, fires an action potential, and then returns back to the rest state. The transition from (a) to (b) is continuous but confined to a very small range of *I* values around *I*_*T*_. At *I* ∼ *I*_*T*_, special solutions called ducks emerge (c), which stay close to the unstable part of the *V*-nullcline, shown as a cubic curve whose attracting branches (resp. repulsive branch) are displayed as solid (resp. dashed) black lines. Two ducks shown in red stay below threshold and correspond to subthreshold oscillations, while one (in purple) crosses threshold and corresponds to a near-threshold spiking solution. Also shown in the top row is the *w* linear nullcline and the equilibrium point (filled circled when stable, open circle when unstable) at the intersection between the 2 nullclines. Single (double) arrows represent fast (slow) flow. Notice that the purple trajectory in panel (c) has the shape of a leftward-directed duck’s profile; see top-right inset. The bottom row shows the time courses of the membrane potential *V* for all trajectories displayed on the top row, keeping the same colors; for panel (c), only the time series of the largest red cycle and of the purple one are shown. Equations and parameter values are given in S1 Text.

Duck solutions have been extensively studied since they were first described in the late 1970s in the context of the van der Pol circuit model [23]. Since then, they have been analyzed in various theoretical and applied contexts, however most of the time within a rather technical mathematical framework. In the next few paragraphs, we present the salient features of canard dynamics in an idealized neural example, the FitzHugh–Nagumo (FHN) spiking model [24,25]. In other words, we present ducks in a nutshell for the general reader, with the accompanying sketch shown in Fig 1; the expert reader may skip the following paragraphs and move directly to page 8. The well-known FHN model describes the generation of action potentials (or spikes) as stable periodic solutions (limit cycles), which exist for a wide timescale separation between the (fast) membrane potential *V* and the (slow) recovery current *w*. Depending on the magnitude of the applied (constant) current *I*, the system’s long-term dynamics is either a stable equilibrium (rest state, shown in panel (a)) or a stable limit cycle (repetitive spiking state, shown in panel (b)). The transition between these 2 neural regimes is rather abrupt in terms of *I*-values. This prompts the following fundamental question: *“How does one understand the very sudden emergence within a small change in I from a stable equilibrium (panel (a)) to a strongly attracting limit cycle (panel (b)) whose trajectory corresponds to sharp/fast transitions from one branch to the other of the V-nullcline*?*”* A 1-parameter family of duck solutions provide a continuous change in solution amplitude over a very small range of *I* values near *I*_*T*_, the value of *I* for which the equilibrium is at the lower “knee” (fold point) of the *V*-nullcline and the system is near Threshold. On top of existing for an extremely narrow range of *I*-values, the essence of ducks is that a portion of the trajectory lies along the repulsive branch of the *V*-nullcline, in the area of the firing threshold. Certain duck solutions stay close to the threshold and then jump down back to baseline (in red in panel (c)), while others jump up and cross threshold while emitting an action potential (in purple in panel (c)). At the end of this transitory regime, a fully developed repetitive spiking solution exists (in blue in panel (b)) and such solution will remain for a large interval of *I*-values greater than *I*_*T*_. This main feature of duck solutions, staying close to the repulsive branch of the *V*-nullcline, hence underlies a common type of threshold behavior in excitable systems of class 2 such as the Hodgkin–Huxley model [18,30]. Furthermore, duck dynamics can be seen in transient responses as well, as part of more complex neural activity (e.g., bursting), where they organize transient passages from subthreshold oscillations to (one or more) spikes. Crucially, duck solutions pass close to special points located at a knee of the *V*-nullcline (see panel (c) where canard cycles pass near the lower knee), linking continuously a zone of dynamical attraction (to the left of the lower knee) to a zone of dynamical repulsion (to the right of the lower knee). In 2D (spiking) models, such a point is called a *canard point*, whereas it is called a *folded singularity* in higher-dimensional models.

**Fig 2:**
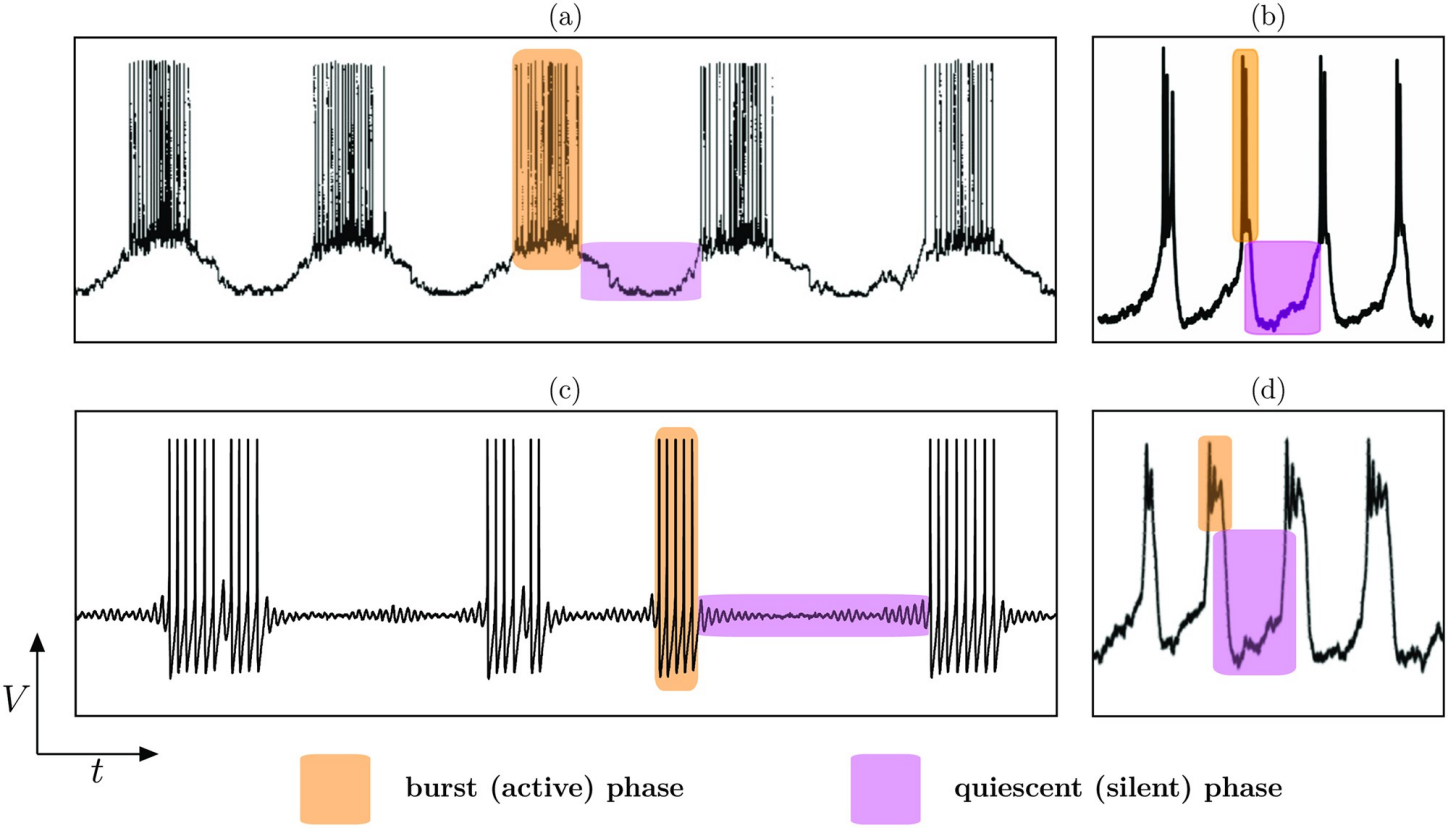
Example of electrophysiological recordings of bursting oscillations in 4 types of neurons: (a) parabolic-type bursting from the CeN neuron from the melibe (a sea slug) [26]; (b) square-wave-type bursting from a human *β*-cell [27]; (c) elliptic-type bursting from a dorsal-root-ganglia (DRG) neuron of a rat [28]; (d) pseudo-plateau-type bursting from a pituitary cell of a rat [29] (“Copyright 2011 Society for Neuroscience”). In each case, we highlight with colors the 2 main phases of bursting oscillations: silent (quiescent) and active (burst). Figures adapted with permission.

Bursting oscillations are ubiquitous in the context of neuronal systems (see Fig 2). In particular, bursting models appeared in the context of classical single-neuron electrophysiological measurements, where the neuron’s voltage time series displays a bursting oscillation either in response to a brief input stimulus or, in absence of any stimuli, in an endogenous manner. These oscillations are defined as having a periodic succession (sometimes irregular) of 2 distinct epochs of activity. One epoch features slow and low-amplitude activity, and it is typically referred to as the *quiescent* (or *silent*) phase. The other epoch features fast and high-amplitude activations (i.e., several action potentials or spikes), and it is classically termed *active* or *burst* phase as shown on several experimental examples in Fig 2.

From the standpoint of multiple timescale models, bursting oscillations require at least 3 dimensions with 2 fast and 1 slow variables, where the 2D *fast subsystem* (obtained by freezing the slow mode and considering it as constant) is bistable within an interval of values of the slow variable (as a parameter) and sustains both stationary and periodic behavior. The quiescent phase corresponds to a slow drift of the solution near a branch of (typically) stationary attractors of the fast subsystem (rest states), and the burst phase to a slow drift with fast oscillatory motion of the solution near a branch of stable limit cycles of the fast subsystem. There can be many ways for a system to produce such alternation between quiescence and burst phases, which motivated scientists early on to develop classification strategies.

In the present work, we will review state-of-the-art classification systems for bursting dynamics, their limitations, and then propose an extended classification framework. Our extended classification rests on the fact that all existing bursting classification schemes are solely based upon the knowledge of the fast subsystem without using the information contained in the *slow subsystem*, obtained when the fast modes have decayed and the associated (fast) variables are slaved to follow the slow variables’ evolution on a limiting phase-space region of slow motion referred to as *critical manifold* [32,33]. One can take advantage of the slow subsystem in order to characterize and classify bursting patterns. Our strategy for doing so relies upon a certain type of canard dynamics, namely that generated by a certain type of folded singularity called *folded node*. In this way, one can extend entire classes of 3D burster to 4D systems, with still 2 fast variables but 1 more slow variable. This second slow variable creates a folded node, near which solutions perform small-amplitude subthreshold oscillations [10], hence enriching the quiescent dynamics of the resulting 4D burster. What is more, the slow subsystem is essential to fully characterize this new type of bursting oscillations with a folded node.

**Fig 3:**
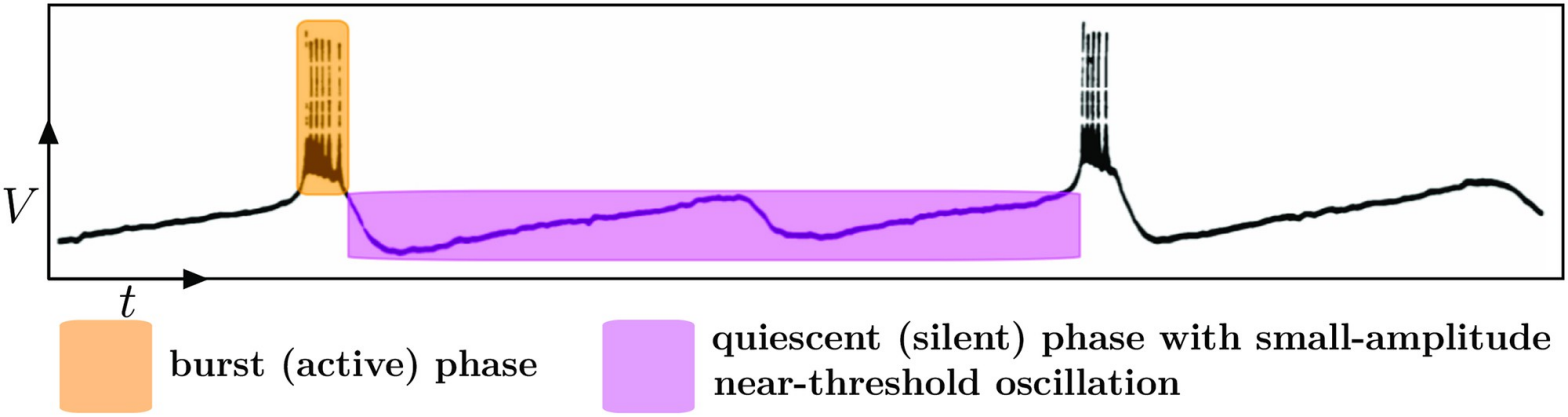
Electrophysiological recordings of the lateral thalamic nuclei neuron in cat from [31] show complex bursting oscillations. Colored boxes highlight the active (burst) and silent (quiescent) phases of the bursting oscillations. The quiescent phase comprises 1 oscillation formed by a slow rise toward bursting threshold and a faster descent toward baseline. This complex bursting pattern is well captured by the “Folded-node/Homoclinic” bursting scenario proposed here. *Figure adapted with permission*.

Slow-fast dynamics near a folded node provide a key mechanism to induce another type of complex oscillations, namely *folded-node-induced mixed-mode oscillations* [10,34]. Hence, the new bursting patterns proposed here are a combination of fold-initiated bursting oscillations (definition given in the next section) and mixed-mode oscillations (MMOs), for which isolated examples were constructed in our previous work [35,36], and which we shall generalize and classify in the present work. This extended framework is well suited to revisit a number of biological datasets where the mechanisms underpinning bursting activity may have not been fully unraveled; see Fig 3 for an example of such data extracted from [31].

This new class of bursting, which we will henceforth denote as *folded-node bursting*, should not be confused with the recent work on so-called “pseudo-plateau bursting”. Initially thought of as a bursting scenario [37], the “pseudo-plateau bursting” case was subsequently shown, in various biophysical models of pituitary cells as well as idealized systems, to be better understood as a MMO mechanism [38,39]. Indeed, the small-amplitude oscillations generated by its folded node were first thought to correspond to a bursting phase. However, since the “pseudo-plateau bursting” involves minimally 2 slow and 1 fast variable, then it does not fall under the bursting definition that demands the fast subsystem to possess 2 fast variables. Hence, these other scenarios (including “pseudo-plateau bursting”) are in stark contrast with the novel folded-node bursting concept presented and classified in the present work, which is the superposition of MMO dynamics and bursting dynamics, with minimally 2 slow and 2 fast variables.

Noteworthy, key to our extended classification are both the fast and slow subsystems. What is more, canards are central to the slow subsystem analysis and, hence, to the classification. In contrast, in the previous classification systems, which only consider the fast subsystem, canards are not useful to the classification. However, they are important to determine certain features of the dynamics, e.g., *spike-adding transitions* [35,43], which will be fully described in the context of system (1), or *torus canards* [44], whose definition and description will be given in the section on cyclic folded-node bursting. Note that *parabolic bursters* [42] require 2 fast and 2 slow variables and possess folded-saddle singularities [45], which makes them different from folded-node bursters.

Although a great deal of our discussions will be in the context of neuronal dynamics, the mathematical framework intends to capture complex slow-fast oscillations beyond the scope of neuroscientific applications (in chemical reactions, genetic switches, material transitions, etc.). Moreover, we will focus on the minimal deterministic mathematical setting for bursting oscillations. This minimal setting will inform more complex scenarios involving multidimensional systems with multiple timescales.

The rest of this article is organized as follows. We will first review existing classification frameworks for bursting oscillations. Subsequently, we will first introduce the key idea of our novel bursting classification based upon the concept of folded-node bursting dynamics. This will be followed by showcasing several new examples of folded-node bursting idealized models, first in the case of *classical* folded node and then in the case of what we will term *cyclic* folded node. We will explain how to construct such bursting dynamics with simple idealized models; for simplicity, however, we will close with a biologically plausible conductance-based model of episodic bursting from [46], showing that the folded-node bursting scenario is applicable and robust to large parameter changes. Finally, in the Conclusions section, we will propose a number of perspectives and future modeling directions worth exploring.

### State-of-the-art classification of bursting patterns

#### Rinzel’s classification (mid-1980s)

**Fig 4:**
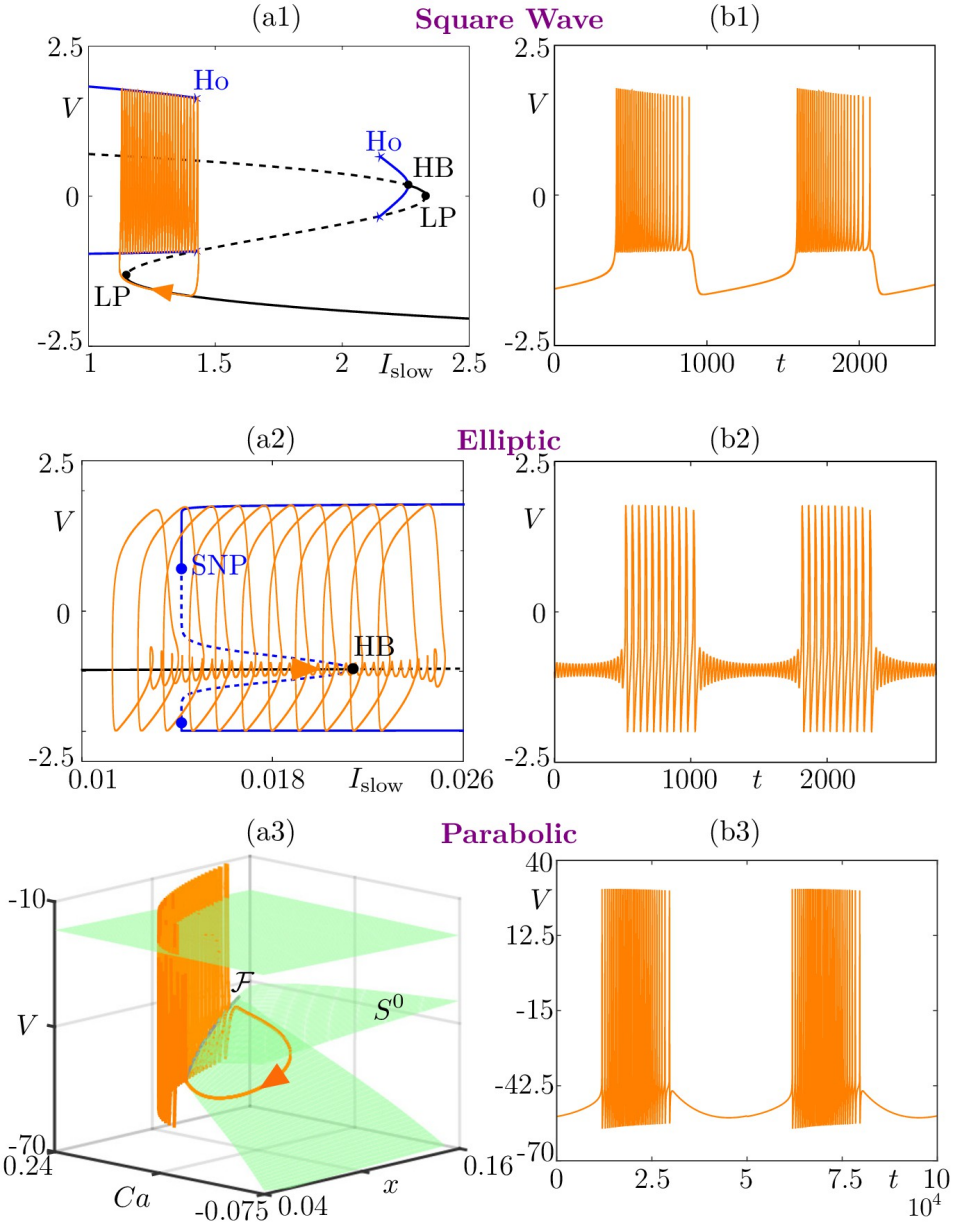
Rinzel classification of bursting patterns. Square-wave bursting, here in the Hindmarsh–Rose model [40] (panels (a1)-(b1)); elliptic bursting, here in the FitzHugh–Rinzel model [6,41] (panels (a2)-(b2)); parabolic bursting, here in Plant’s model [42] (panels (a3)-(b3)). In each case, we show a phase-space projection of the bursting solution of the full system (orange) together with the bifurcation diagram of the fast subsystem (left panel) and the time course of membrane potential *V* (orange, right panel). Labels for bifurcations are as follows: Ho for homoclinic, HB for Hopf bifurcation, LP for saddle-node (limit point) bifurcation of equilibria, and SNP for saddle-node of periodic orbits. (a3) The *critical manifold S*^0^ (green) is the *S*-shaped (not fully rendered) surface of equilibria of the fast subsystem; this surface is folded along the *fold curve* F. Equations and parameter values are given in S1 Text.

Historically, the second author of the present work opened the door toward mathematically understanding bursting oscillations. His seminal work on a mathematical analysis and classification of bursting oscillatory patterns was first published within 2 companion manuscripts [6,41]. The fundamental insight behind Rinzel’s classification is based on so-called *slow-fast dissection* and, in particular, on describing the bifurcation structure of the fast subsystem where the slow variables are frozen. Subsequently, the time trajectory of the full system (i.e., for small *ε* > 0) is superimposed on top of the bifurcation structure of the fast subsystem. This is the essence of slow-fast dissection, which reveals that the quiescent phase of the bursting cycle corresponds to trajectory segments where the solution slowly tracks families of stable equilibria, or low-amplitude (subthreshold) limit cycles, of the fast subsystem. Conversely, the burst phase of the full system’s cycle corresponds to trajectory segments where the solution slowly tracks families of limit cycles of the fast subsystem. Crucially, the transitions between these 2 main phases of bursting cycles occur near bifurcation points of the fast subsystem. With this approach, Rinzel proposed 3 classes of bursting dynamics based on both the bifurcation structure of the fast subsystem and the salient features of the main fast variable’s time profile (in the neuronal context, this is typically the neuronal membrane potential). These features include spike frequency during the burst, dynamics during the silent phase (oscillatory or not), shape of the burst (on a plateau compared to the silent phase or, on the contrary, with undershoots). These 3 features led Rinzel to name 3 classes: *square-wave bursting*, observed in a number of recordings and models of pancreatic beta-cells [47] among other [48]; *elliptic bursting*, observed in various neural recordings and models of sensory neurons [15,49]; and *parabolic bursting*, initially observed in the Aplysia R15 neuron [42] and ever since in various neural models [50]. We show an example of each class in Fig 4.

#### Izhikevich’s classification (ca. 2000)

**Fig 5:**
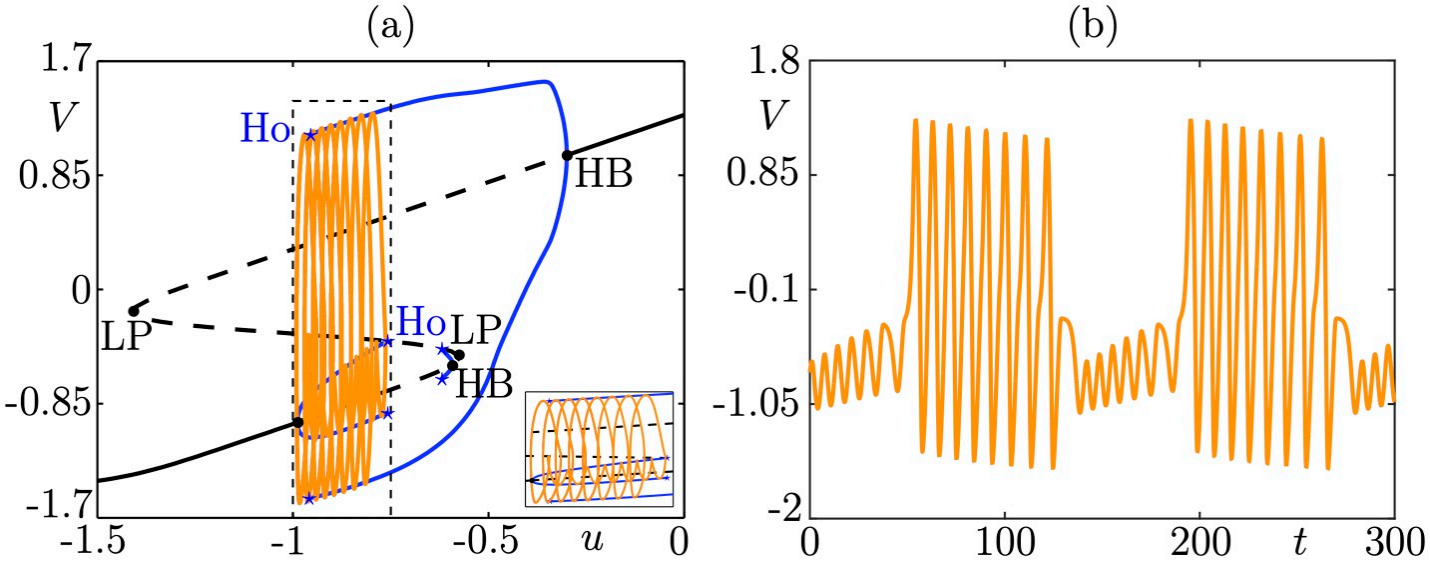
Small homoclinic/big homoclinic bursting, corresponding to Fig 88 of [7]; shown is a new simulation with the same parameter values (available in [7]). Panel (a) shows the slow-fast dissection in the (*u*, *V*) phase plane; the inset shows a zoom corresponding to the dashed rectangle, which better reveals the shape of the bursting cycle while the main plot better highlights the fast subsystem bifurcation structure. Labels HB, LP, and Ho refer to Hopf bifurcation, saddle-node bifurcation (fold or “limit point”), and homoclinic bifurcation of the fast subsystem, respectively. Panel (b) shows the *V*-time series of this bursting solution. Izhikevich’s classification allowed to characterize bursting patterns beyond square-wave, elliptic and parabolic, and already opened the door toward explaining more complex biological data. In particular, one can mention pathological brain activity related to, e.g., epileptic seizure [52] or spreading depolarization [16,53]. According to Izhikevich’s classification, bursting oscillations where the burst initiates via a fold bifurcation of the fast subsystem are termed *fold-initiated bursting*. In the present work, we will propose an extended classification based upon fold-initiated bursting cases.

Eugene Izhikevich generalized Rinzel’s approach by considering that a bursting pattern is entirely characterized by a pair of bifurcations (**b**_**1**_, **b**_**2**_) of the fast subsystem. One bifurcation, say **b**_**1**_, explains the transition from quiescence to burst, and the other, **b**_**2**_, marks the inverse transition, from burst to quiescence. Due to the well-established bifurcation theory and indeed knowledge of classes of bifurcation, this led to a systematic identification of at least 120 bursting patterns [7]. An example of a bursting model that is not within the Rinzel classification is depicted in Fig 5. In this example, the bursting pattern has a transition from quiescence to burst via a homoclinic bifurcation (involving a small homoclinic connection ending a family of small-amplitude limit cycles), and, equally, the transition from burst to quiescence is via homoclinic bifurcation (involving a large homoclinic connection ending a family of large-amplitude limit cycles). In many ways, Izhikevich’s work serves as a key source of reference for classification of complex slow-fast oscillations. This is particularly the case in neuroscience since some of the assembled examples were motivated by existing conductance-based neuronal models and demonstrated how complex neuronal oscillations could be achieved by adding 1 slow equation to a spiking model. Indeed, a dedicated book toward neuroscience was later published, where the derived models where also put into context with neurophysiological processes [51]. The result of this deeply insightful work is a quasi-complete classification of bursting patterns in terms of pairs of codimension-one bifurcations of the fast subsystem.

#### Bertram and colleagues’s/Golubitsky and colleagues’s classification (mid-1990)

An alternative approach to classification was proposed by Bertram and colleagues in 1995 [8] and extended mathematically by Golubitsky and colleagues in 2001 [9] using a singularity theory viewpoint. The fundamental idea consists in identifying a codimension-*k* bifurcation point (*k* ≥ 2) in the fast subsystem and subsequently considering the slow variables of the bursting system as the unfolding parameters of this high codimension bifurcation point. The bursting is then obtained via a path made by the slow variables in the unfolding of that point (i.e., within a multidimensional parameter space). The minimum codimension, whose unfolding allows to create a given bursting pattern, defines the class of the associated bursting patterns provided a notion of path equivalence is properly defined. Specifically, 2 paths are equivalent if one can pass from one to the other via a diffeomorphism and a reparameterization. Recently, a review and a showcase demonstrating the construction of bursting oscillations via this approach, including cases for higher codimension bifurcation points was proposed in [54].

It is worth noting that the Rinzel–Izhikevich approach and the Bertram–Golubitsky approach both focus on the fast subsystem only. Moreover, a way to see a link between the 2 approaches is that the 2 bifurcation points (**b**_**1**_, **b**_**2**_) of the fast subsystem (as characterized by Izhikevich’s approach) belong to bifurcation curves in a 2-parameter plane, which coalesce at a codimension-two bifurcation point that characterizes this particular bursting pattern from the singularity theory viewpoint. This implies that, in principle, the Rinzel–Izhikevich and the Bertram–Golubitsky approaches both lead to the same number of bursting oscillation cases. Finally, one can consider more complicated slow paths in the fast subsystem’s parameter space, which may induce more than 2 crossings of bifurcation curves; see, e.g., [55]. However, this will likely not increase the number of possible bursting patterns captured.

The bursting patterns covered by these 3 existing classification schemes have not been exhausted yet, even though a large number (way above 100) have already been reported and analyzed in previous works. We have identified a few more cases, which we believe have not been considered before and which are presented in idealized models in an earlier version of the present work [56]. In particular, we show scenarios where the burst phase ends due to a transcritical or a pitchfork bifurcation of limit cycles of the fast subsystem. We also propose the concept of *isola bursting*, where the burst starts and ends through fold-of-cycles bifurcations lying on an isola of limit cycles. Finally, we propose 1 example (among many) of bursting pattern with 2 slow variables where the burst initiates through a family of transcritical bifurcation of equilibria.

As a side note, we mention hybrid models like integrate-and-fire models, including both ODEs and reset maps, which are able to produce bursting oscillations as well as canard-induced spike-adding phenomena; see, e.g., [57–61]. To the best of our knowledge, there is no classification of bursting patterns in these models, which might involve additional mechanism due to the nonsmooth nature of the equations. However, bursting patterns in fully discrete neural models, i.e., maps, have been classified in [62] using the classical fast subsystem approach.

### Extended classification: Folded-node bursting

#### Going beyond the state-of-the-art

There are bursting oscillations beyond the Rinzel–Izhikevich and Bertram–Golubitsky classification approaches and which cannot be explained by invoking these classical results. We propose an extended classification system that captures a larger class of bursters beyond state-of-the-art approaches.

Indeed, some electrophysiological recordings of bursting dynamics resist the state-of-the-art classification system. A case in point is depicted in Fig 3, where the bursting oscillation has 2 phases but the quiescent phase is peculiar: It rises twice per period close to a threshold, however the first time the neuron does not transit to the active phase and instead descends back to its baseline activity, while the second time only the active phase emerges.

These observations suggest that there is an underlying complex mechanism for the quiescent phase of the oscillations and therefore point toward a bursting classification framework that has to also incorporate the analysis of the slow subsystem, which is in stark contrast to state-of-the-art approaches. Further motivating this view is our earlier study [35], in which we constructed what seems to be the first example of a slow-fast bursting model whereby the burst initiation could not be explained by the fast subsystem of the underlying model; we constructed another example in [36]. However, therein, we did not attempt to derive an improved bursting classification framework and it is what we are now proposing.

**Fig 6:**
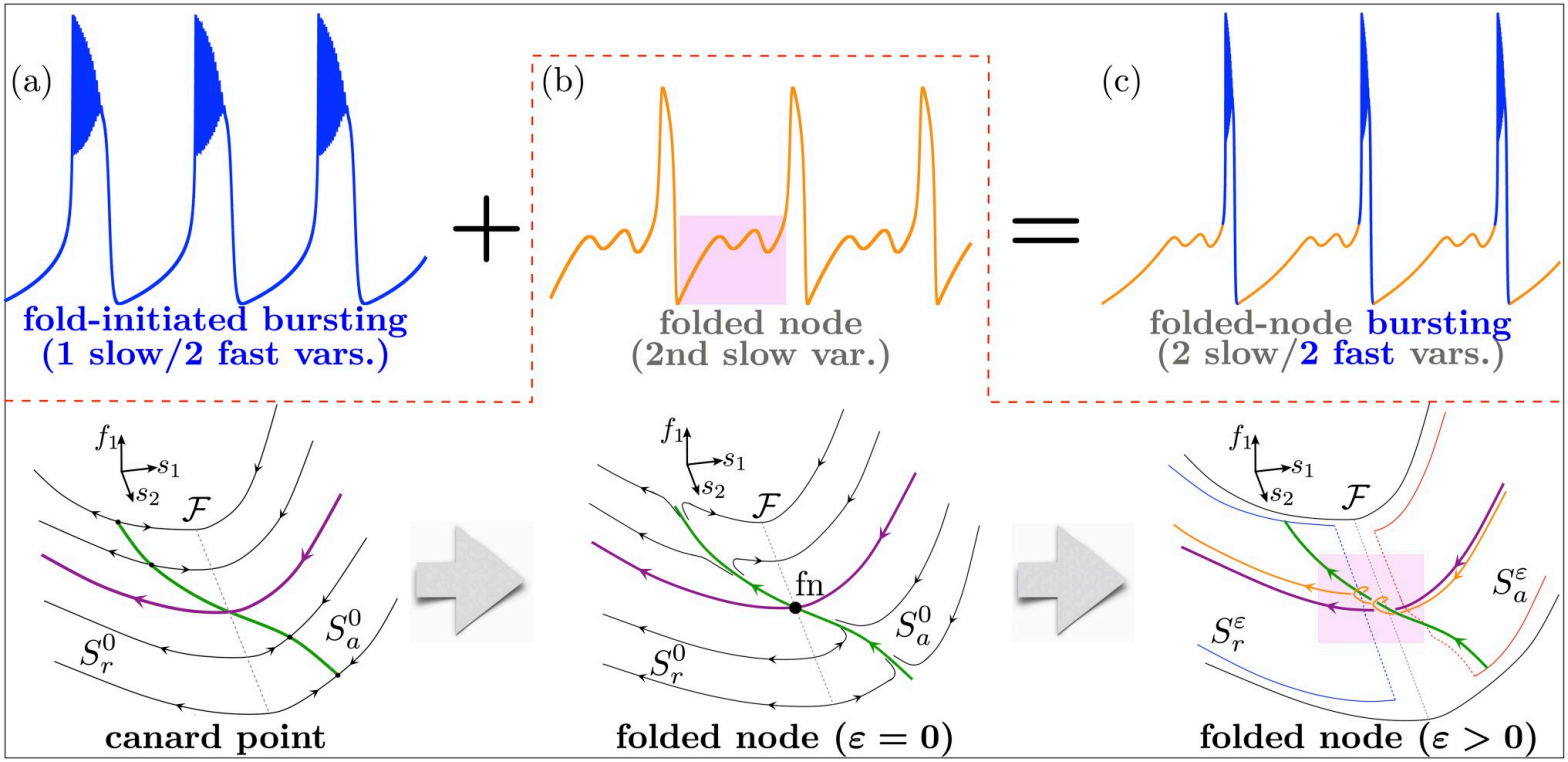
Folded-node bursting in a nutshell. The top row shows the essentials of folded-node bursting: (a) A fold-initiated bursting system (*f*_1_, *f*_2_, *s*_1_) (*f*_1,2_ are fast and *s*_1_ is slow) with (b) an added slow variable *s*_2_ creating a folded node and corresponding to the main parameter of the 3D burster organizing spike-adding transitions gives (c) a 2 slow variables/2 fast variables folded-node burster. The bottom row is an extension of the top panel (b) and shows the essentials of folded-node dynamics (whose typical time course is shown in the top panel (b)): A canard point (*ε* = 0) in the (*f*_1_, *f*_2_, *s*_1_) bursting system with *s*_2_ as parameter (left panel) becomes a folded node (black dot, center panel, *ε* = 0) when the slow dynamics put on *s*_2_ is evolving, for *ε* = 0 along the attracting and repelling parts $S_{a,r}^{0}$ of the critical manifold; for small *ε* > 0, this folded node creates small-amplitude oscillations nearby, organized by attracting and repelling slow manifold $S_{a,r}^{\varepsilon }$ (perturbations of $S_{a,r}^{0}$) and responsible for the quiescent oscillations of the folded-node burster in the resulting 4D system. See S1 Text for a glossary of labels and technical terms.

We will show in subsequent sections how to construct a variety of these new cases of bursting oscillations. To further motivate and guide the reader throughout this manuscript, we first delineate the main mechanisms underpinning our extended classification framework. The idea is sketched in Fig 6 and can be summarized in its simplest form as follows.

If one considers any 3D fold-initiated burster and appends to it a second slow variable that organizes (as a bifurcation parameter in the original burster) a spike-adding transition, then one obtains a new bursting system with 2 slow and 2 fast variables, for which the bursting pattern can only be fully characterized by using both slow and fast subsystems. Indeed, due to the added second slow variable, the novel burster possesses subthreshold oscillations, which are due to the presence of a folded-node singularity defined in the slow subsystem (*ε* = 0) and associated canard solutions, which persist for small enough *ε* > 0.

**Fig 7:**
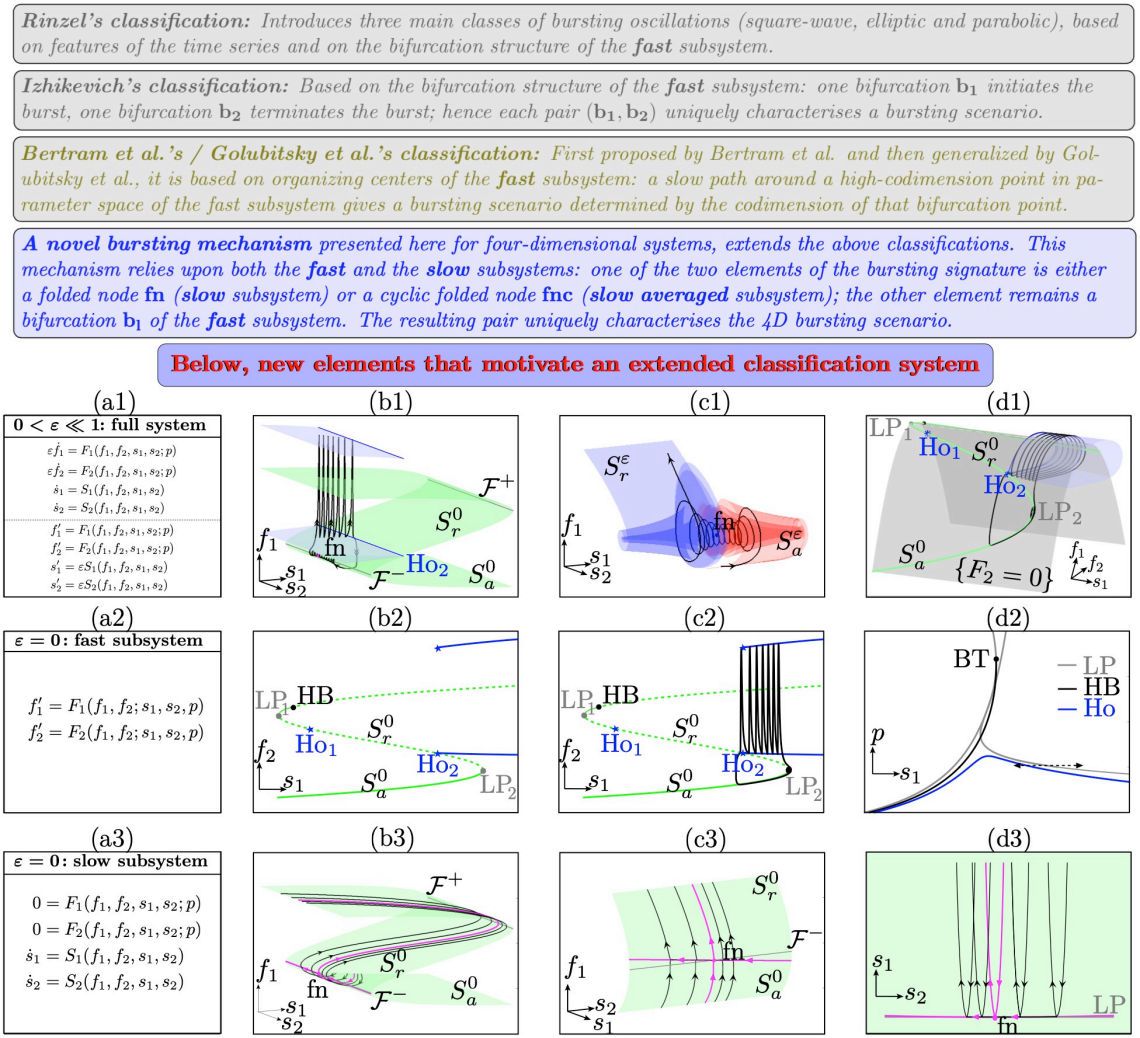
Extended classification. **(Top part)** Main idea of the Rinzel/Izhikevich, Bertram/Golubitsky and colleagues and folded-node bursting classifications. **((ai)-(di), i = 1,2,3)** Exemplary “folded-node/homoclinic” bursting, presented in the full 4D system and in its 2D fast and slow subsystems (resp.), showing that both subsystems are required to fully understand this bursting profile; all equations are given in the left column (a1)-(a3). **Top row (b1)-(d1), full system bursting solution** in 2 different 3D phase-space projections: 2 slow/1 fast in (b1)-(c1) and 1 slow/2 fast in (d1), also showing the critical manifold (fast subsystem’s set of equilibria, in green), the fast subsystem’s limit cycles envelope (blue), as well as relevant bifurcations. In (c1), the trajectory is zoomed near its small oscillations, which follow attracting (red) and repelling (blue) slow manifolds $S_{a,r}^{\varepsilon }$, perturbations of the attracting and repelling parts $S_{a,r}^{0}$ of the critical manifold, and pass near the folded node (dot). **Middle row (b2)-(d2), fast subsystem information**: the bifurcation diagram with respect to 1 slow variable (*s*_1_) in (b2), which we can assume persists as such for a small interval of values of the other slow variable (*s*_2_); this allows to superimpose the projection of the full system bursting orbit (c2), as done in the Rinzel/Izhikevich classification, and to compute loci of bifurcation points of this diagram in the 2-parameter plane (*s*_1_, *p*), as done in the Bertram/Golubitsky and colleagues classification. However, both approaches classify this bursting pattern as fold/homoclinic (square-wave), hence failing to capture the reason for its small oscillations during quiescence, which can only be unraveled by studying the **slow subsystem’s information in the bottom row (b3)-(c3)** and find the existence of a folded node in the slow singular limit; details on labels in S1 Text.

How this type of bursting effectively extends the previous classifications is summarized in Fig 7. Crucially, the folded node due to the second slow variable is not a bifurcation of the fast subsystem even though it lies on a curve of saddle-node (fold) bifurcations of the fast subsystem (see Fig 7 panel (d3)). Such unexpected and nontrivial emergent mathematical objects allow trajectories of the slow subsystem to visit both the attracting ($S_{a}^{0}$) and repelling ($S_{r}^{0}$) parts of the critical manifold. In the full system (for small *ε* > 0), the perturbed versions of these manifolds—attracting $S_{a}^{\varepsilon }$ and repelling $S_{r}^{\varepsilon }$ slow manifolds [32,33]—twist and intersect multiple times (see Fig 7 panels (b2)-(c2)), thereby causing trajectories to nontrivially and robustly oscillate during the quiescent phase of the bursting system.

In essence, a folded node appears when the slow dynamics at *ε* = 0 change direction along a fold curve. In the full system, the transition from quiescent to active phase is caused by a repulsion of the trajectory away from the unstable sheet of the critical manifold; this phenomenon is mediated by folded-node canards. In this particular example, the fast oscillations of the active phase are due to a nearby Hopf bifurcation in the fast subsystem (not shown). The return back to quiescence is then caused by a family of homoclinic bifurcations (labeled Ho_2_ in panels (b1), (d1), (b2), (c2)) of the fast subsystem.

The key insight is that the fast subsystem is blind to what is causing these small-amplitude oscillations during the quiescent phase, and thus it is unable to classify the initiation of these oscillations based upon the bifurcations of fast subsystem only. This point is illustrated by the Rinzel–Izhikevich slow-fast dissection and projection of the trajectory of the full system onto the bifurcation diagram of the fast subsystem (see Fig 7 panels (b2)-(c2)).

Note that by employing the Rinzel–Izhikevich classification system, the bursting dynamics would be explained by 2 bifurcations of the fast subsystem, namely the fold bifurcation LP_2_ and the homoclinic bifurcation Ho_2_. In particular, a fold bifurcation (LP_2_) does not explain an oscillation. Moreover, a similar argument applies to the Golubitsky approach (see Fig 7 panel (d2)). This panel displays curves of codimension-one bifurcation points of the fast subsystem, which meet at codimension-two, e.g., a Bogdanov–Takens (BT) (within a 2D parameter space). It can then be shown that it is impossible to construct a path for the slow dynamics (within this 2D parameter space), in particular along the homoclinic and saddle-node curves (since these characterize the bursting in the fast subsystem), which could explain folded-node-initiated quiescent phase oscillations.

It turns out that among all possible folded singularities, only folded nodes (and in limiting cases, so-called *folded saddle-nodes*) can generate such robust small-amplitude oscillations in the full system, and this is due to the twisting of nearby attracting and repelling slow manifolds. This leads us to a novel bursting classification system (see Fig 7 top panel in blue for the new framework). We believe that these insights will fuel subsequent developments in higher-dimensional multiscale systems.

This underlying folded-node signature leads us to name the resulting new classes of bursting models, folded-node bursters. Three fundamental cases are envisaged. The first case is bursters characterized by small-amplitude oscillations that occur during the quiescence phase, in which case we will refer to the *classical folded-node* bursting scenario. The second case involves slow-amplitude modulation of the burst, which we will denote as the *cyclic folded-node* bursting scenario. We use the term cyclic folded node since it corresponds to having a folded singularity on a line of cyclic fold bifurcations of the fast subsystem, whereas the classical folded node corresponds to having a folded singularity on a line of (stationary) fold bifurcations of the fast subsystem). The third case combines *classical folded-node* and *cyclic folded-node*. These classes of bursting patterns involve both the fast subsystem and the slow subsystem of the model, unlike traditional bursters. A second key aspect of these new classes is the central role played by *canards*, namely, *spike-adding canard* cycles involved in the classical folded-node bursting case, and *torus canards* in the cyclic folded-node bursting case. In the following subsections, we describe in details these 2 scenarios.

#### Classical folded-node bursting case

**Fig 8:**
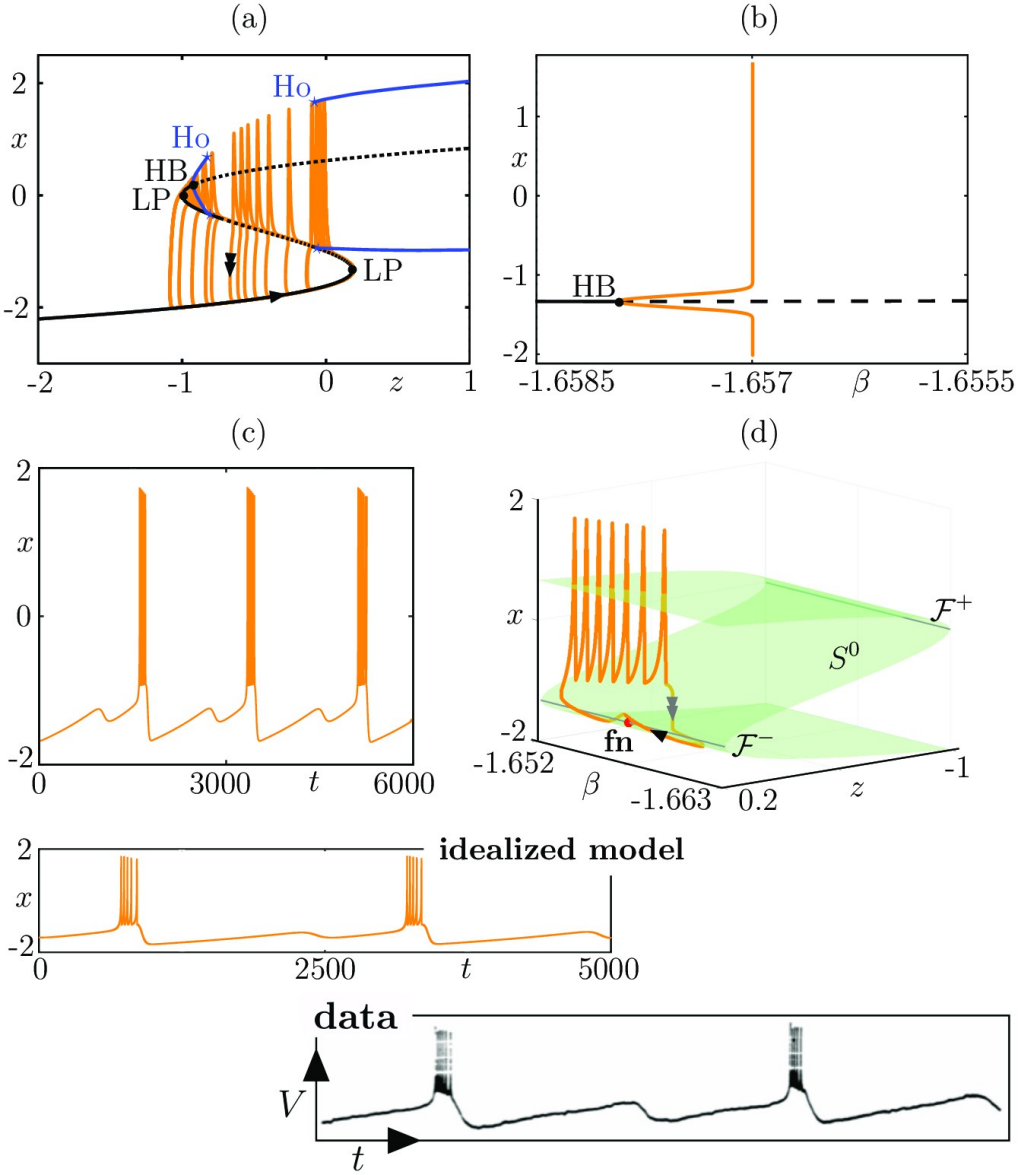
Folded-node/Homoclinic bursting. Panels (a-b) show the spike-adding transition in system (1): (a) in the (*z*, *x*) plane where we show several limit cycles for *β*-values exponentially close to −1.656996 superimposed onto the fast subsystem bifurcation diagram; (b) bifurcation diagram of the associated 3D bursting system (1) with respect to parameter *β*, showing the sharp rise of the amplitude of the limit cycle branch (orange), corresponding to spike-adding transitions. Panels (c-d) show a folded-node/homoclinic bursting orbit in the extended 4D system (6): (c) in the (*β*, *z*, *x*) space (single/double arrows indicate slow/fast motion); (d) time course of the fast variable *x*. The bottom panels show a comparison between this folded-node bursting orbit from (6) and experimental data from [31]. Equations and parameter values are given in S1 Text.

**Fig 9:**
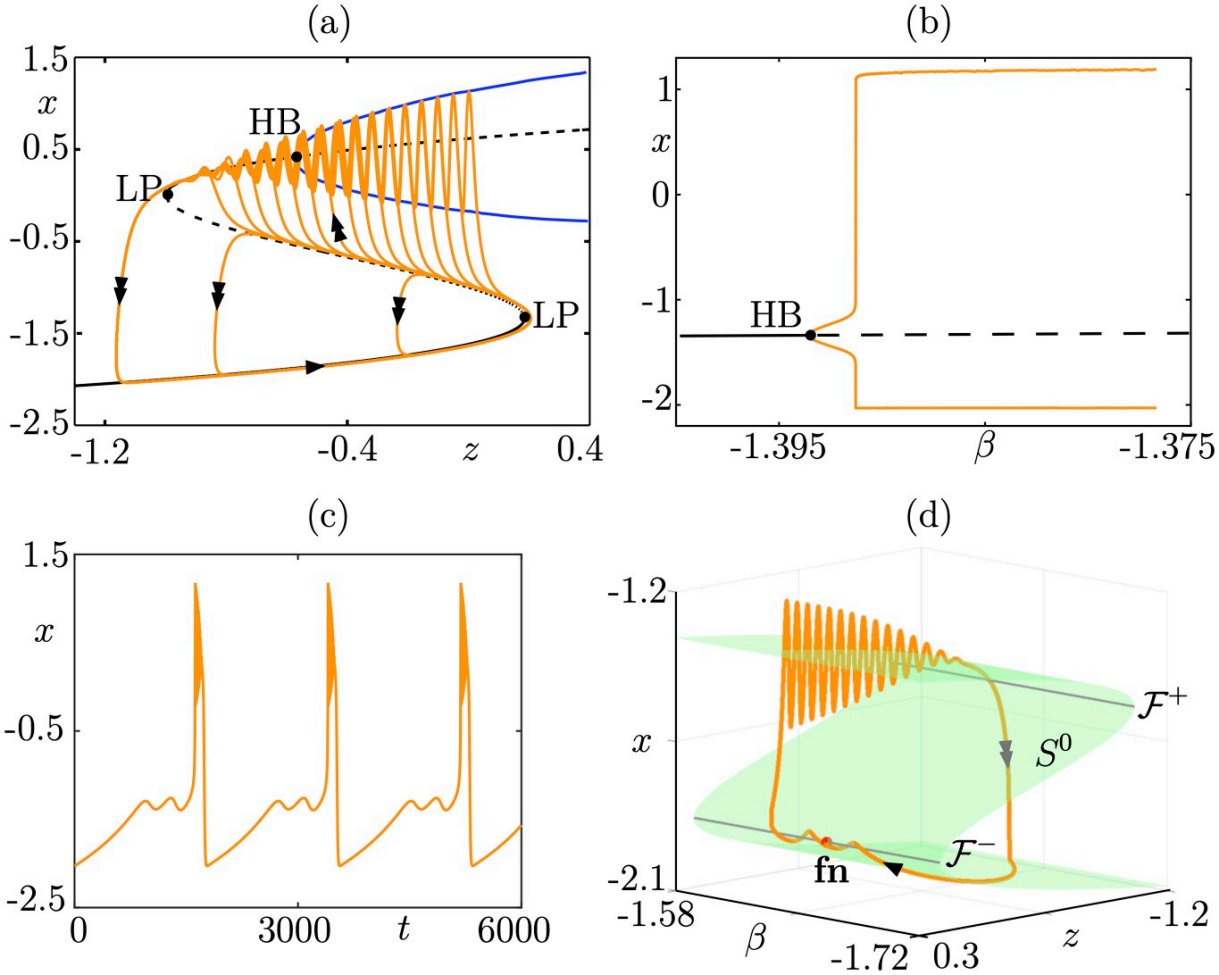
Folded-node/Hopf bursting. Panels (a-b) show the spike-adding transition in system (1): (a) in the (*z*, *x*) plane where we show several limit cycles for *β*-values exponentially close to −1.391279 superimposed onto the fast subsystem bifurcation diagram; (b) bifurcation diagram of the associated 3D bursting system (1) with respect to parameter *β*, showing the sharp rise of the amplitude of the limit cycle branch (orange), corresponding to spike-adding transitions. Panels (c-d) show a folded-node/Hopf bursting orbit in the extended 4D system (6): (c) in the (*β*, *z*, *x*) space (single/double arrows indicate slow/fast motion); (d) time course of the fast variable *x*. Equations and parameter values are given in S1 Text.

**Fig 10:**
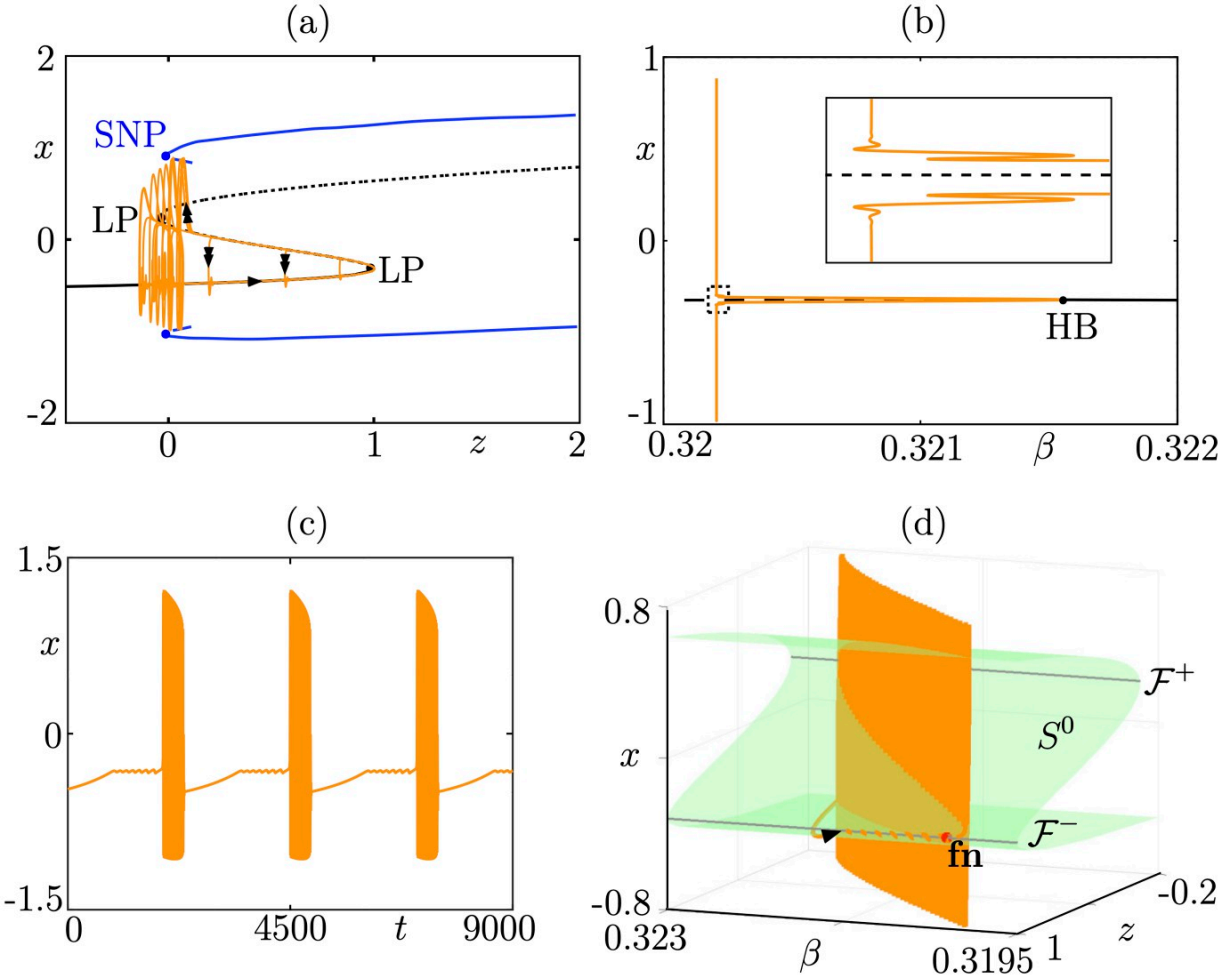
Folded-node/fold-of-cycles bursting. Panels (a-b) show the spike-adding transition in system (1): (a) in the (*z*, *x*) plane where we show several limit cycles for *β*-values exponentially close to 0.320207 superimposed onto the fast subsystem bifurcation diagram; (b) bifurcation diagram of the associated 3D bursting system (1) with respect to parameter *β*, showing the sharp rise of the amplitude of the limit cycle branch (orange), corresponding to spike-adding transitions. Panels (c-d) show a folded-node/fold-of-cycles bursting orbit in the extended 4D system (6): (c) in the (*β*, *z*, *x*) space (single/double arrows indicate slow/fast motion); (d) time course of the fast variable *x*. Equations and parameter values are given in S1 Text.

Here we propose several bursting oscillations mediated by a classical folded node (fully described in Figs 8, 9 and 10) and the modeling steps of underlying idealized models are given. To guide the reader toward a modeling strategy of these systems, we first recall key concepts and mechanisms.

#### A necessary preliminary step: Spike-adding canard explosion

First, recall that *canards* are nontrivial trajectories that emerge due to timescale separation and unexpectedly, these trajectories contain segments that follow both an attracting slow manifold and a repelling slow manifold, which are perturbations of attracting and repelling branches of the fast nullcline, respectively; see Fig 1(c). This phenomenon has been thoroughly studied in planar systems (i.e., with 1 slow variable and 1 fast variable) [23,63–65], as well as in 3D systems with 2 slow variables [10,66,67].

In applications, canards can be associated to complex (bio)physical mechanisms, e.g., in neuroscience, it provides the best approximation to the excitability threshold in certain single-neuron models. This observation was first made by Izhikevich [51], who showed that canards organize the transition to the spiking regime of type II neurons. This was later analyzed in more details in [21,22,68].

Another important mechanism is the so-called *spike-adding canard explosion*. This canard phenomenon arises in bursting oscillations and can be described as a sequence of canard explosions, which organize the transition from subthreshold oscillations to bursting solutions with more and more spikes per bursts. This phenomenon was first described and analyzed (in the case of chaotic dynamics regime) in [69] in the context of square-wave bursting. This was revisited more recently in [70] from the computational standpoint of saddle-type slow manifolds and further described in [71] in a modeling context to explain transient spikes; see also [72,73]. These analyzes were later refined (from a canard standpoint) in [35], and the canard-mediated spike-adding dynamics was fully analyzed in [45] in the context of parabolic bursters (with 2 slow variables), revealing the central role of folded-saddle canards.

Noteworthy, bursting oscillations that possess a spike-adding mechanism correspond to a limiting (borderline) case that already hints at the importance of possibly including the analysis of the slow flow (flow of the slow subsystem; see below) in a bursting classification framework. That is, spike-adding requires a turning point of the slow flow (canard point), whereby each new added spike (within the bursting phase) is born via a slow (delayed) passage through this turning point. Crucially, the fast subsystem is blind to the underlying canard trajectories occurring near the turning point (well defined as such only in the slow flow) and instead only sees a fold bifurcation. Therefore, the state-of-the-art bursting classification systems does not capture this aspect. Nevertheless, we refrain from declaring this phenomenon as a new bursting mechanism because a spike-adding canard explosion gives rise to canard cycles that exist only within exponentially thin parameter regions. Hence, the robust dynamics is the fold-initiated bursting dynamics, and the fast subsystem analysis still prevail in order to classify it.

In contrast, if we consider a fold-initiated bursting scenario undergoing spike-adding canard explosion and if we further add a slow dynamics for the parameter that displays the spike-adding canard explosion (i.e., a second slow variable in the extended model), then we obtain a folded-node bursting system. This has a similar effect to the case in classical (van der Pol type) systems where the canard phenomenon becomes robust if one adds a second slow variable, which has the effect of creating a folded singularity in the resulting 2D slow flow and allows for multiple canard trajectories to exist. The idea here is similar, but with 2 fast variables, allowing for bursting dynamics in conjunction with folded-node dynamics.

A first example of this scenario was termed *mixed-mode bursting oscillations* in [35], but we prefer to denote it more generally folded-node bursting. Indeed, folded-node bursting is a new form of bursting pattern with 2 slow variables where the silent phase contains small-amplitude (subthreshold) oscillations due to the presence of a folded node in the slow subsystem. This folded node is responsible for the presence of a funnel region in the full system and trajectories entering this funnel make a number of rotations (which can be controlled by adjusting parameters) before they leave it and start to burst. Hence, the passage through the folded-node funnel organizes the transition from quiescence to burst and it can only be understood by suitably analyzing the slow subsystem. We subsequently describe a strategy for constructing folded-node bursting systems.

#### Construction of minimal folded-node bursting systems

As a starting point, we consider the prototypical fold-initiated burster of Hindmarsh–Rose type [40]. By this, we mean a 3D slow-fast system with 2 fast variables and 1 slow and a cubic-shaped family of equilibria in the fast subsystem, namely the critical manifold *S*^0^. We can write the following set of differential equations (using the so-called *fast time τ*) to describe the dynamics of such a system:

$$\begin{array}{ll} x' & =y-f(x)+az,\\ y' & =G(x,y,z),\\ z' & =\varepsilon (\alpha x+\gamma \beta -\delta z), \end{array}$$ (1)

where *f* is a cubic polynomial function, *G* is (at least) quadratic in *x*; moreover, 0 < *ε* ≪ 1 is a small parameter and (*a*, *α*, *β*, *γ*, *δ*) are potential bifurcation parameters; why we use a product of 2 parameters in the *z* equation will become clear below. As we shall see in the example of Fig 10, one can also obtain all fold-initiated scenarios by using an unfolding of a codimension-3 degenerate BT bifurcation; see [74] for details.

A few assumptions are required in order for system (1) to display fold-initiated bursting. First of all, we assume that *f* and *G* are adequately chosen so that the fast subsystem has a cubic-shaped family of equilibria that depends on *z* as a parameter (for the fast subsystem). Therefore, the corresponding bifurcation diagram (of the fast subsystem) in *z* is S-shaped and will have fold points. The critical manifold is then given by

$$S^{0}≔\left\{(x,y,z)\in R^{3}\;/\;G(x,y,z)=0,\;z=\left(f(x)-y\right)/a\right\}.$$ (2)

We also require bistability in the fast subsystem between equilibria and limit cycles, in an interval of *z*-values. One bound of this interval correspond to a fold bifurcation and, geometrically, to 1 fold point of the cubic family of equilibria *S*^0^. The other boundary of the region of bistability of the fast subsystem will be a bifurcation of limit cycles, and we shall consider 3 main cases, namely, saddle-homoclinic bifurcation (see Fig 8(a)), Hopf bifurcation (see Fig 9(a)), and fold bifurcation of cycles (see Fig 10(a)); the list is not exhaustive, we chose to focus on these 3 examplary cases; however, more examples of folded-node bursting scenarios can be constructed by following the procedure highlighted here and by choosing a different bifurcation of the fast subsystem ending the burst. Now, considering the linear slow dynamics of system (1) for the slow variable *z*, we assume that a variation of one of the 2 parameters *α* and *β* in the full system induces the linear *z*-nullsurface to cut through the fold point of the critical manifold *S*^0^ for a certain value of this parameter. One can show that this creates a Hopf bifurcation in the full system, which induces limit cycles to appear. Provided this transversal cut of the *z*-nullsurface with the critical manifold takes place, then a spike-adding canard explosion will emerge, whereby bursting solutions appear from subthreshold (spikeless) periodic solutions along branch of limit cycles undergoing multiple canard explosions; see [35] for an example of this spike-adding phenomenon in the context of square-wave bursting. In Figs 8, 9 and 10 panels (a), we show the standard slow-fast dissection of 3D fold-initiated bursters of the type of system (1), with several limit cycles of the 3D bursting system (orange curves) within the spike-adding regime (with respect to parameter *β*) are superimposed onto the fast subsystem bifurcation diagram (which does not depend on the value of *β*). In panel (b) of each figure, we show the bifurcation diagram of the 3D burster (1) with respect to parameter *β*, where the sharp rise of the (orange) branches of limit cycles (born at the Hopf bifurcation, labeled HB, and indicated by a black dot) indicates spike-adding canard explosions that organize the transition from the stationary to the bursting regime.

As explained in the previous section, one salient feature of the spike-adding canard explosion is the presence of a turning point (a canard point) in the slow flow of system (1). To compute this slow flow, we first rescale time in (1) by a factor *ε*. That is, we rescale the fast time *τ* (with *x*′ = *dx*/*dτ*) into the so-called *slow time t* defined by *t* = *ετ*. This brings the system to the slow-time parametrization

$$\begin{array}{ll} \varepsilon \dot{x} & =y-f(x)+az,\\ \varepsilon \dot{y} & =G(x,y,z),\\ \;\dot{z} & =\alpha x+\gamma \beta -\delta z, \end{array}$$ (3)

whose *ε* = 0 limit corresponds to the slow subsystem, also called the *reduced system*. The slow subsystem is a differential-algebraic equation (DAE), where the dynamics of *z* is explicitly preserved while *x* and *y* are slaved to *z* by the algebraic constraints that corresponds to the Eq (2) of the (here 1D) critical manifold *S*^0^. The dynamics of *x* and *y* can be revealed by differentiating the algebraic constraint with respect to the slow time *t*, which gives after rearranging the following 1D dynamical system defined on *S*^0^

$$\begin{array}{ll} \dot{x} & =\frac{\left(aG_{y}-G_{z})(\alpha x+\gamma \beta -\delta z\right)}{G_{x}+G_{y}f'(x)}, \end{array}$$ (4)

where *G*_*p*_ is the partial derivative of *G* with respect to *p* ∈ {*x*, *y*, *z*} and *f*′(*x*) is the derivative of *f* with respect to *x*. As is typical in slow-fast systems with folded critical manifolds, note that the denominator of the right-hand side of (4) vanishes at fold points of *S*^0^ (defined by the condition det(J_(*x*,*y*)_) = 0 where J_(*x*,*y*)_ is the Jacobian matrix of (3) with respect to the fast variables (*x*, *y*)), which makes generically the dynamics of *x* explode at the corresponding fold point, referred to as a *jump point*. However, if the numerator has a zero of the same order as the denominator, then there can be a cancellation and the dynamics of *x* does not explode; in this case, the fold point is referred to as a canard point or a turning point. The condition for a canard point to occur in this system is then given by

$$\begin{array}{ll} z_{f} & =(\alpha x_{f}+\gamma \beta )/\delta , \end{array}$$ (5)

where (*x*_*f*_, *z*_*f*_) is a fold point of *S*^0^ and assuming *aG*_*y*_ − *G*_*z*_ ≠ 0 as a nondegeneracy condition. This indeed gives a transversal crossing of the slow nullsurface with the critical manifold at one of its fold points. Even though (5) depends on several parameters, it is a codimension-one condition, therefore by fixing all parameters but one, then the condition can be satisfied by adjusting the last parameter. We arbitrarily choose to vary *β*, which will become a second slow variable in the full 4D folded-node bursting system that we will construct below. Therefore, the spike-adding transitions leading to bursting in system (1) are obtained as the result of the slow nullsurface moving though 1 fold point of the critical manifold upon variation of *β*.

The same dynamics would be obtained by varying a parameter affecting the critical manifold while maintaining the slow nullsurface fixed, in particular if we were to append an additive parameter *I* to the *x* equation of the system. This would mimick the effect of an applied (external) current in a neuron-type model such as the Hindmarsh–Rose model [40] or the Morris–Lecar model [69,75]. However, from the pure dynamical viewpoint, varying a parameter in the slow equation results in the same effect and this is the scenario that we chose in order to construct fold-initiated spike-adding transitions in the original 3D burster and folded-node bursting in the extended 4D model.

Starting from a fold-initiated bursting scenario with spike-adding canard explosion (controlled via a static variation of parameter *β*), a folded-node bursting is then obtained by prescribing the dynamics on *β* by a slow differential equation. That is, we consider the following extended bursting system

$$\begin{array}{ll} x' & =(y-f(x)+az)/c,\\ y' & =G(x,y,z),\\ z' & =\varepsilon (\alpha z+\gamma \beta -\delta x),\\ \beta ' & =\varepsilon (\mu -\gamma_{y}(y-y_{f}{)}^{2}-\gamma_{\beta }(\beta -\beta_{f}{)}^{2}). \end{array}$$ (6)

For suitable choices of the additional parameters *μ*, *γ*_*y*_, *y*_*f*_, *γ*_*β*_ and *β*_*f*_, we can obtain folded-node bursting dynamics in the resulting 4D system (6), of the type dictated by the underlying bursting in the (*x*, *y*, *z*) system. Then, in panels (c) of Figs 8, 9 and 10, we show the time course for the *x* fast variable of the ensuing folded-node/homoclinic, folded-node/Hopf, folded-node/fold-of-cycles bursting orbits, respectively. We observe, as expected, that the burst part looks very similar to that of the underlying 3D fold-initiated bursting system; however, the quiescent part has small-amplitude oscillations due to the second slow variable *β* that creates a folded node; see below. The folded-node bursting dynamics is further showcased in panel (d) of Figs 8, 9 and 10, where we show it (orange curve) in the (*β*, *z*, *x*) 3D projection of the 4D phase space together with the 2D critical manifold *S*^0^ of the full system (green *S*-shaped surface), its 2 fold curves $F^{\pm }$ and the folded-node point lying on the lower fold curve $F^{-}$, labeled fn and indicated by a black dot. The critical manifold *S*^0^ and the folded node fn are only obtained through the slow subsystem (singular slow limit *ε* = 0) and are key to fully characterize these 3 bursting patterns, which in the classical classification systems would be termed exactly like their underlying 3D burster. Two additional panels are given in Fig 8 to show how this idealized folded-node/homoclinic model can reproduce experimental data that do not match any bursting pattern in the previous classification systems. Note that our idealized model was not initially designed to explain these data from [31] (also displayed in Fig 3), yet the time profiles match remarkably well. The strong similarity between our idealized model and these data suggest that folded-node bursting constructions could potentially inform the design of biophysical models.

Note that we consider here prototype systems (6) where either *G* is directly given as a graph over *x*, described as, *G*(*x*, *y*, *z*) = *g*(*x*) − *y* (i.e., Folded-node/homoclinic, in Fig 8, and folded-node/Hopf cases, in Fig 9), or the level set {*G*(*x*, *y*, *z*) = 0} is a graph over (*x*, *z*), expressed as {*y* = *g*(*x*, *z*)} (i.e., Folded-node/fold-of-cycles case, in Fig 10). We claim that all folded-node initiated bursting scenarios can be obtained in either of these 2 ways. In the latter case, our minimal model is inspired by the codimension-3 degenerate Bogdanov–Takens unfolding introduced in [74] and further applied in the context of bursting in [54].

In practice (for simulation purposes), *μ*, *γ*_*y*_, and *γ*_*β*_ will be taken *O*(*ε*). Therefore, system (6) is effectively a 3-timescale dynamical systems with dynamics evolving on *O*(1), *O*(*ε*), and *O*(*ε*^2^) timescales. For convenience and to ease the folded-node analysis, we will keep the equations written as in (6) with only *ε* has an apparent timescale separation parameter.

Introducing the slow time *t* = *ετ* brings system (6) into the parametrization

$$\begin{array}{ll} \varepsilon \dot{x} & =(y-f(x)+az)/c,\\ \varepsilon \dot{y} & =G(x,y,z),\\ \dot{z} & =\alpha z+\gamma \beta -\delta x,\\ \dot{\beta } & =\mu -\gamma_{y}(y-y_{f}{)}^{2}-\gamma_{\beta }(\beta -\beta_{f}{)}^{2}, \end{array}$$ (7)

whose *ε* = 0 limit corresponds to the slow subsystem. We will show that, all other parameters being fixed, the slow subsystem of (7) possesses a folded-node singularity, which creates transient subthreshold oscillations that initiate the burst when 0 < *ε* ≪ 1, regardless of the values of other parameters.

However, simulations require that *μ γ*_*y*_ and *γ*_*β*_ be *O*(*ε*) in order for these small subthreshold oscillations to be recurrent, hence entering into a robust periodic bursting attractor, which we name folded-node bursting. We provide numerical evidence of this point, based on the strength of the global return mechanism, even though we do not provide a rigorous proof of it.

Applying the same strategy as in the 3D (bursting) case, and projecting onto the (*x*, *β*)-plane (the dimension of the slow flow corresponds to the number of slow variables), we obtain the following equations for the reduced system (slow subsystem)

$$\begin{array}{ll} \dot{x} & =\frac{\left(g_{z}(x,z)+a)(\alpha z+\gamma \beta -\delta x\right)}{f'(x)-g_{x}(x,z)},\\ \dot{\beta } & =\mu -\gamma_{y}{\left(g(x,z)-y_{f}\right)}^{2}-\gamma_{\beta }(\beta -\beta_{f}{)}^{2}, \end{array}$$ (8)

after substituting for *g*(*x*, *z*) for *y* from the critical manifold condition. The critical manifold of system (6) is not *normally hyperbolic* [32] (loosely speaking, it means that fast subsystem equilibria are hyperbolic) everywhere and, hence, the system possesses a (1D here) fold set defined by

$$F≔\{(x,y,z)\in S^{0};\;f'(x)=g_{x}(x,z)\}.$$

This implies that the slow flow (8) of system (6) is not defined along F. The slow flow can be extended along F by performing an *x*-dependent time rescaling, which amounts to multiply the right-hand side of (6) by a factor *f*′(*x*) − *g*_*x*_(*x*, *z*), hence yielding the so-called *desingularised reduced system (DRS)*

$$\begin{array}{ll} \dot{x} & =\left(g_{z}(x,z)+a\right)(\alpha z+\gamma \beta -\delta x),\\ \dot{\beta } & =\left(f'(x)-g_{x}(x,z)\right)\left(\mu -\gamma_{y}{\left(g(x,z)-y_{f}\right)}^{2}-\gamma_{\beta }(\beta -\beta_{f}{)}^{2}\right), \end{array}$$ (9)

with *z* = *z*(*x*) defined by *S*^0^, that is, *g*(*x*, *z*) − *f*(*x*) + *αz* = 0. In all cases, we will consider (including the general codimension-3 unfolding of a degenerate BT bifurcation from [74]), *z* can be written as a function of *x* on *S*^0^. As a consequence of this *x*-dependent time rescaling, the DRS (9) is regular everywhere in $R^{2}$ including on F, along which it has the possibility for equilibria simply by appearance of the factor *f*′(*x*) − *g*_*x*_(*x*, *z*) in the *β*-equation.

The equilibrium condition is then that $\dot{x}=0$ in (9) together with *f*′(*x*) − *g*_*x*_(*x*, *z*) = 0, which conveys an idea already seen in the 3D (bursting) case. That is, a singularity of the reduced system at a point on F can be resolved if and only if the numerator of the right-hand side of $\dot{x}$ in that system vanishes at this point and the zeros of the 2 algebraic expressions to be of the same order. Such points are called *folded singularities* (or *folded equilibria*), and they are the equivalent of canard points in the cases with (at least) 2 slow variables.

Folded equilibria are equilibria of the DRS (9) and, according to their topological type as equilibria of the DRS, one can generically define *folded nodes*, *folded saddles*, and *folded foci*. However, they are not equilibria of the reduced system (8) due to the singular time rescaling performed to pass from one to the other. Indeed, this time rescaling is chosen so that trajectories of the DRS have reversed orientation on the repelling sheet of *S*^0^ compared to trajectories of the reduced system (both have the same orientation along the attracting sheet). Hence, in the case of folded nodes and folded saddles, trajectories starting on the attracting sheet of *S*^0^ may cross the folded singularity in finite time and with finite speed, which is not possible with an equilibrium.

The Jacobian matrix of (9) evaluated at a folded equilibrium has the form

$$\text{J}=\left(\begin{array}{cc} \left(-\delta +\alpha z^{'}(x))(g_{z}(x,z)+a\right) & \gamma (g_{z}(x,z)+a)\\ K_{2} & 0 \end{array}\right)$$ (10)

where

$$K_{2}=(f''(x)-g_{xx}(x,z))\left(\mu -\gamma_{y}{\left(g(x,z)-y_{f}\right)}^{2}-\gamma_{\beta }(\beta -\beta_{f}{)}^{2}\right).$$

From (10), one can easily write down conditions that enable the emergence of a folded-node singularity (tr(*J*) < 0, det *J* > 0, tr(*J*)^2^ − 4 det *J* > 0) or a folded-saddle singularity (det *J* < 0) in the reduced system.

As we will explain below, even though only the folded-node case gives rise to robust bursting patterns, the folded-saddle case is still interesting in the study of 4D bursters with 2 slow variables. One also can easily verify that our minimal example systems all give rise to a folded-node case. Indeed, in the folded-node/homoclinic (Fig 8) and folded-node/Hopf (Fig 9) bursting cases, system (6) has the form

$$\begin{array}{ll} x' & =(y-x^{3}+3x^{2}+z)/c,\\ y' & =1-5x^{2}-y,\\ z' & =\varepsilon (\alpha z+\gamma \beta -\delta x),\\ \beta ' & =\varepsilon \left(\mu -\gamma_{y}(y-y_{f}{)}^{2}-\gamma_{\beta }(\beta -\beta_{f}{)}^{2}\right), \end{array}$$ (11)

which hence gives the following DRS’s Jacobian matrix

$$\begin{array}{l} J_{1,2}=\left(\begin{array}{ll} -\delta & \gamma \\ K_{2} & 0 \end{array}\right), \end{array}$$ (12)

with: $K_{2}=(-6x_{fs}-4)\left(\mu -\gamma_{y}{\left(1-5x_{fs}^{2}-y_{f}\right)}^{2}-\gamma_{\beta }(\beta -\beta_{f}{)}^{2}\right)$, and *x*_fs_ = −4/3.

Given the chosen parameter values corresponding to Figs 8 and 9, then we immediately conclude that we have indeed a folded node. Likewise, in the folded-node/fold of cycles case illustrated in Fig 10, the slow-fast system corresponding to (6) is

$$\begin{array}{ll} x' & =y,\\ y' & =-x^{3}+A_{1}(z)x+A_{2}(z)-y(A_{3}(z)-x+x^{2}),\\ z' & =\varepsilon (\alpha z+\gamma \beta -\delta x),\\ \beta ' & =\varepsilon \left(\mu -\gamma_{y}(y-y_{f}{)}^{2}-\gamma_{\beta }(\beta -\beta_{f}{)}^{2}\right), \end{array}$$ (13)

where *A*_*i*_ = *a*_*i*_*z* + *b*_*i*_ (*i* = 1,2,3) are linear functions of *z*. Therefore, we obtain the associated DRS’s Jacobian matrix

$${\text{J}}_{1,2}=\left(\begin{array}{cc} \left(-\delta +\alpha \frac{3x_{\text{fs}}^{2}-b_{1}}{a_{1}+a_{2}})(a_{1}x_{\text{fs}}+a_{2}\right) & \gamma_{\beta }{\left(a_{1}x_{\text{fs}}+a_{2}\right)}^{2}\\ K_{2} & 0 \end{array}\right),$$ (14)

with: $K_{2}=(6x_{fs}-a_{1})\left(\mu -\gamma_{y}y_{f}^{2}-\gamma_{\beta }(\beta -\beta_{f}{)}^{2}\right)$ and *x*_fs_ solution to

$$-3x_{fs}^{2}+a\frac{x_{fs}^{3}-b_{1}x_{fs}-b_{2}}{a_{1}+a_{2}}+b_{1}=0.$$

Substituting the parameter values for their chosen numerical value mentioned in the caption of Fig 10 allows to conclude that we are indeed dealing with a folded node.

One can obtain the general DRS (9) by applying implicit differentiation to 1 algebraic equation only (the right-hand side of the $\dot{x}$ equation in the original system) and substituting *g*(*x*, *z*) for *y* (coming from the second algebraic equation). This gives the same result as the DRS obtained from both algebraic constraint together. Indeed, in all generality, applying implicit differentiation to the 2 algebraic equations of the slow subsystem gives

-f'(x)1-gx(x,z)1x˙y˙=-agz(x,z)(αz+γβ-δx)z˙=αz+γβ-δx,β˙=μ-γy(y-yf)2-γβ(β-βf)2, (15)

which by Kramer’s rule is equivalent, after posing

$$J=\left(\begin{array}{ll} -f'(x) & 1\\ -g_{x}(x,z) & 1 \end{array}\right),$$

(Jacobian matrix of the original vector field with respect to the fast variables at *ε* = 0) to

$$\begin{array}{ll} det(J)\left(\begin{array}{l} \dot{x}\\ \dot{y} \end{array}\right) & =Adj(J)\left(\begin{array}{l} \;a\\ -g_{z}(x,z) \end{array}\right)(\alpha z+\gamma \beta -\delta x)\\ \dot{z} & =\alpha z+\gamma \beta -\delta x,\\ \dot{\beta } & =\mu -\gamma_{y}(y-y_{f}{)}^{2}-\gamma_{\beta }(\beta -\beta_{f}{)}^{2}, \end{array}$$ (16)

where det(J) = *g*_*x*_(*x*, *z*) − *f*′(*x*) and

$$\text{Adj(J})=\left(\begin{array}{cc} 1 & -1\\ g_{x}(x,z) & -f^{'}(x) \end{array}\right),$$

denote the determinant and the adjugate matrix of J, respectively. The previous system is singular when det(J) vanishes, which happens on the fold set. It can be desingularized by rescaling time by a factor det(J), which brings the DRS in its most general form, namely

$$\begin{array}{ll} \left(\begin{array}{l} \dot{x}\\ \dot{y} \end{array}\right) & =Adj(J)\left(\begin{array}{l} \;a\\ -g_{z}(x,z) \end{array}\right)(\alpha z+\gamma \beta -\delta x)\\ \;\dot{z} & =det(J)(\alpha z+\gamma \beta -\delta x)\\ \;\dot{\beta } & =det(J)(\mu -\gamma_{y}(y-y_{f}{)}^{2}-\gamma_{\beta }(\beta -\beta_{f}{)}^{2}). \end{array}$$ (17)

After being projected onto the (*x*, *β*)-space, system (17) then takes the form

$$\begin{array}{ll} \dot{x} & =(a+g_{z}(x,z))(\alpha z+\gamma \beta -\delta x)\\ \dot{\beta } & =(f'(x)-g_{x}(x,z))(\mu -\gamma_{y}(y-y_{f}{)}^{2}-\gamma_{\beta }(\beta -\beta_{f}{)}^{2}), \end{array}$$ (18)

which indeed agrees with (9).

With the above analysis, we can construct in principle any folded-node burster of our liking. We showcase 3 examples in Figs 8, 9 and 10: folded-node/homoclinic bursting, folded-node/Hopf bursting, and folded-node/fold-of-cycles bursting, respectively.

Finally, we quickly reflect on why folded-saddle bursting is not robust. The folded-saddle case is simply a different parameter regime in the slow subsystem; however, the resulting dynamics is substantially different than that generated by a folded node. In neuron models with (at least) 2 slow variables, folded saddles and their associated canard solutions play the role of firing threshold. In particular, in the context of bursting system, they have recently been shown to organize the spike-adding transition in parabolic bursters [45,76]. Counterintuitively, small-amplitude oscillations can also emerge in the vicinity of a folded saddle; see [77] for a rigorous analysis of this phenomenon and also [11,76] for further related work. However, there is no funnel near a folded saddle and the canard dynamics is hence not robust, which applies no matter how many fast variables the system possesses, so in particular in the context of bursting. This is why, in systems with (at least) 2 fast and 2 slow variables, only the folded-node case gives rise to a new class of bursting oscillations.

#### Cyclic folded-node bursting case

In the same spirit as in the classical folded-node case, one can construct interesting bursting rhythms where the slow oscillations occur on the envelope of the burst and this is due to what we will denote *cyclic folded node*. Parallel to the construction of a folded-node burster system, one can construct a cyclic-folded-node burster system by considering a 3D slow-fast system, which possesses *torus canard* solutions.

Loosely speaking, torus canard corresponds to a canard phenomenon with a fast rotation. Already mentioned by Izhikevich in [78] in a canonical model, it was later found in a biophysical model of Cerebellar Purkinje cell exhibiting fold/fold cycle bursting [44] and subsequently analyzed with more mathematical details in, e.g., [79,80]. Even though to date not all elements of torus canard transitions have been mathematically unraveled, one can summarize this phenomenon by emphasizing that its key feature corresponds to a canard explosion within a fast oscillatory motion. Instead of slowly following a family of equilibria past a fold bifurcation, the fast-oscillating system slowly follows a family of limit cycles past a cyclic fold bifurcation.

One can draw a parallel between classical canards and torus canards in their role of transitional regime in neuronal dynamics: Classical canards can explain the rapid transition from rest to the spiking regime, likewise torus canards can explain the rapid transition from the spiking to the bursting regime. Furthermore, torus canards are also not robust and only exist within exponentially thin parameter regions.

Thus, the very same idea that leads from canard point to folded singularities can lead from torus canard to cyclic folded-node canards, when adding a second slow variable. In this way, a cyclic folded-node can be robust even if the torus canards are not robust. This has been proposed very recently by Vo and colleagues [81,82] via a specific example that links the resulting dynamics to the amplitude-modulated bursting already mentioned in [44,78]; see also [83–85] for other examples of amplitude-modulated bursting.

**Fig 11:**
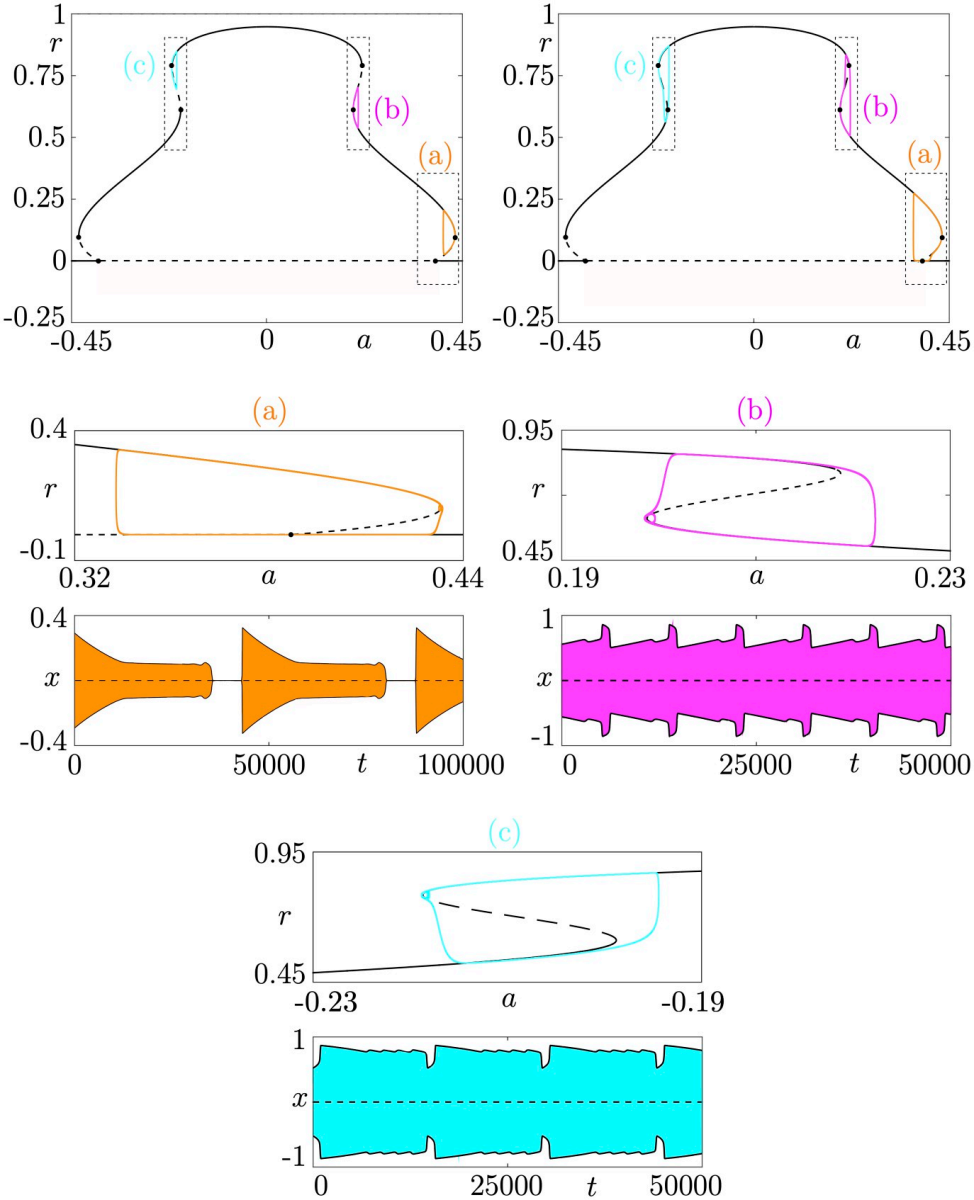
Cyclic folded-node bursting cases. We use polar coordinates in order to construct idealized models. The top panels show the slow-fast dissection for the amplitude variable *r* of the underlying bursting model, with 3 different torus canard scenarios (a), (b), and (c). Adding a slow dynamics on a parameter *β* controlling the slow nullcline then yields associated cyclic folded-node bursting scenarios for which we show both the slow-fast dissection in the (*a*, *r*) plane and the *x* time series: (a) initiated by a subcritical Hopf bifurcation; (b) terminated by a fold of cycles; (c) initiated by a fold of cycles. Equations and parameter values are given in S1 Text.

In summary, we herein propose a taxonomy of cyclic folded-node bursting patterns, with several numerical examples, which completes our extension of the previous bursting classifications. We complement this with a few examples of idealized models displaying cyclic-folded-node bursting. We consider systems expressed in polar form, in which case the condition for cyclic folded node and then for cyclic folded-node bursting reduce to (classical) folded-node conditions on *r*; see Fig 11. We start with a bursting system written in (*r*, *θ*, *a*) coordinates and displaying torus-canard dynamics, the type of which depends on the location of the slow nulllcline in the original bursting system, assuming for simplicity that this slow nullcline is horizontal of the form {*r* = *β*}. Then, we put a slow dynamics on *β* similar to the one in system (6), which yields cyclic-folded-node bursting dynamics.

**Fig 12:**
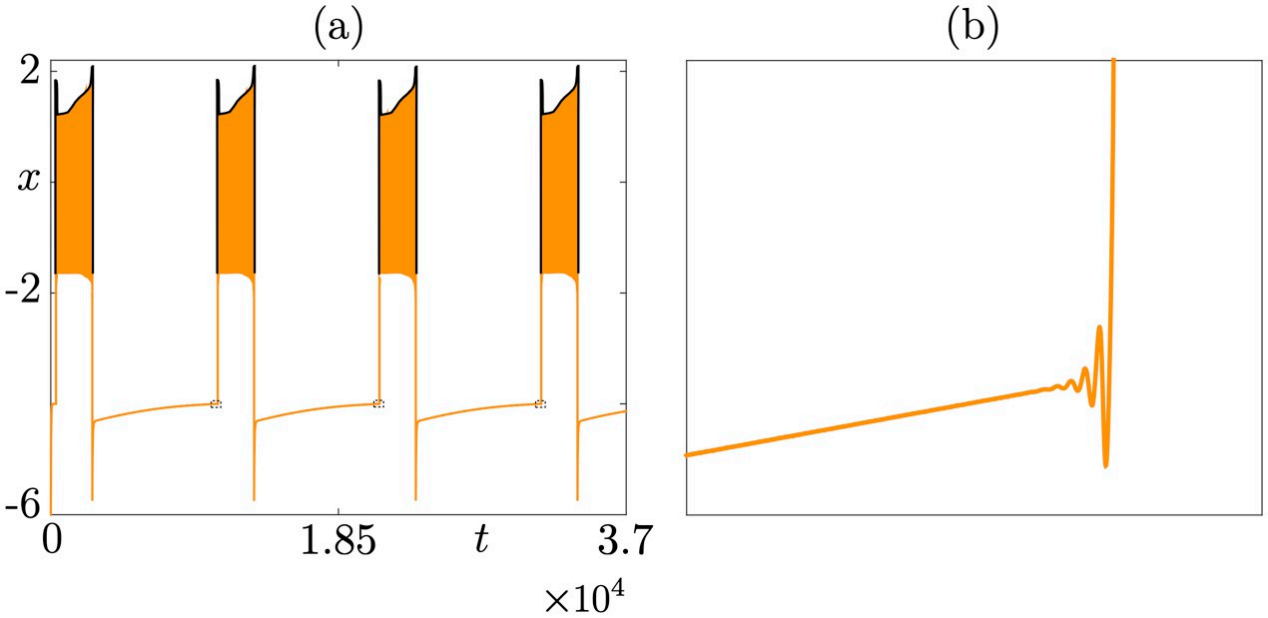
A folded-node/cyclic folded-node bursting example. The *x*-times series of the folded-node/cyclic folded-node bursting solution is shown in panel (a), where the upper envelope of the burst phase has been traced in black in order to better show the small-amplitude oscillations of this envelope due to the presence of a cyclic folded node; panel (b) is a zoom of panel (a) near the classical folded node highlighting small-amplitude oscillations throughout the silent phase. Equations and parameter values are given in S1 Text.

In general, it is possible to reduce the system locally near the cyclic fold bifurcation of the fast subsystem enabling the computation of normal form coefficients (see [81,82,84,85]) that effectively characterize the cyclic folded-node. However, the bursting conditions have not been established in general. Finally, for sake of completeness, we construct a limiting case of a nontrivial system that displays both classical folded-node bursting and cyclic-folded-node bursting, as depicted in Fig 12.

### Application to conductance-based models

**Fig 13:**
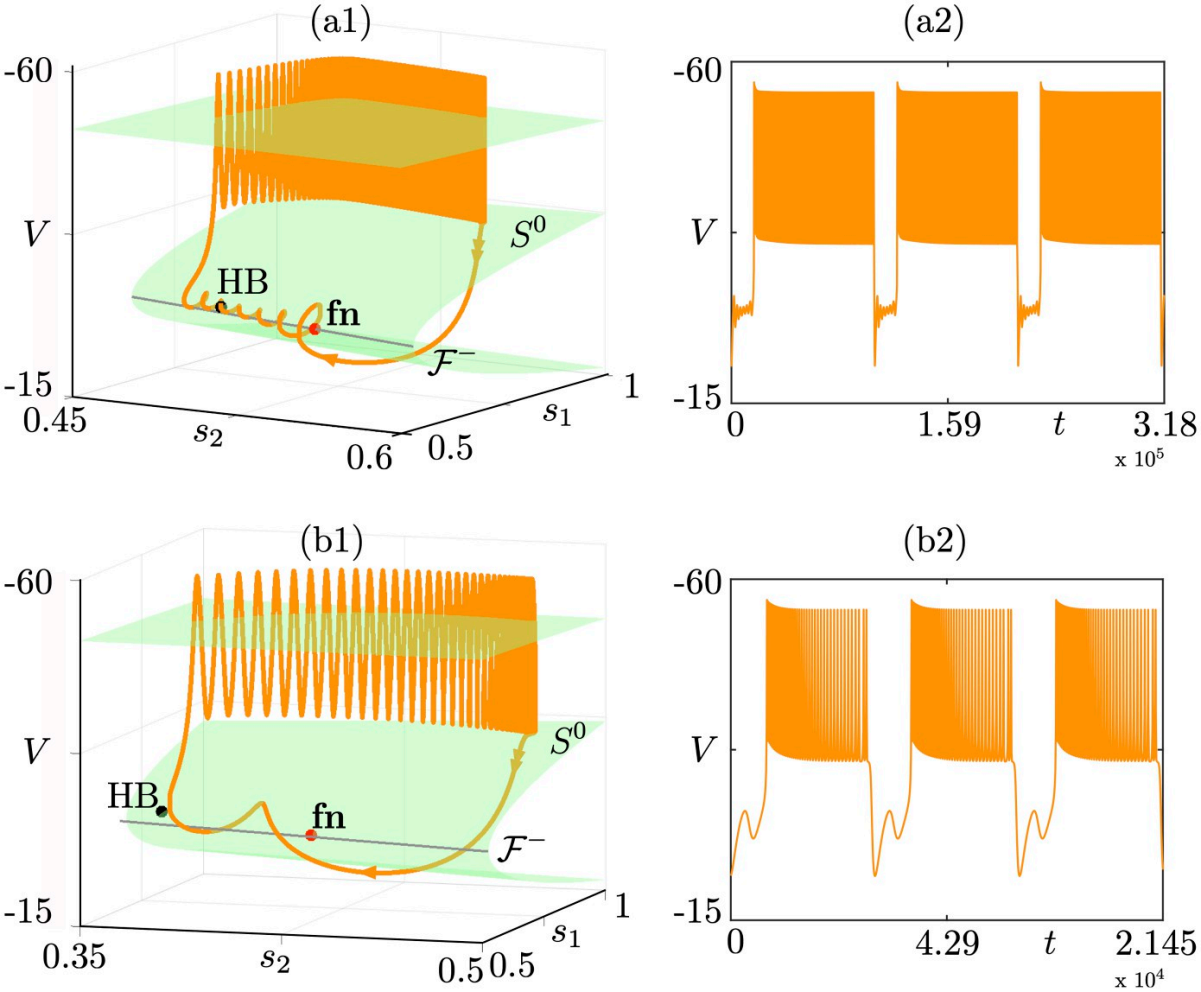
A conductance-based episodic bursting example [46]. Left panels: Folded-node bursting orbits shown in the (*s*_1_, *s*_2_, *V*)-space projection together with the 2D critical manifold *S*^0^, the lower fold curve $F^{-}$, the folded-node singularity **fn**; we also show the location of the Hopf bifurcation point (HB) of the 3D fast subsystem assuming only *s*_2_ as a slow variable. Right panels: *V* time series. The top panels show a bursting orbit for the original parameter values from [30], whereas the bottom ones show a similar bursting solution for a different parameter set where only the kinetics of the 2 slow currents have been modified. In the second parameter set, the HB point moves out of the subthreshold oscillation region and, hence, the one-slow-variable scenario does not fully explain the bursting pattern, which is better cast as folded-node bursting. The parameters of the slow currents that we modify to obtain the new set are as follows: $g_{K_{1}}=18.5,\;g_{K_{2}}=20,\;\tau_{s_{1}}=600,\;\tau_{s_{2}}=4000,\;v_{s_{1}}=-51,\;v_{s_{2}}=-35$. All equations and parameter values are given in S1 Text.

We now provide a biophysical example, namely a conductance-based bursting model, without explicit timescale separation and which we show can be recast as a folded-node burster. This model is a so-called *episodic burster* that was introduced by Bertram and colleagues in [46] to model beta-cell oscillations, known to produce square-wave type bursting patterns. Noteworthy, this model contains 4 state variables, 2 being fast—the membrane potential *V* and the delayed rectifier potassium current activation *n*—and 2 slow—activation variables *s*_1_, *s*_2_ corresponding to 2 additional potassium currents—as described in [46]. The system’s equations read

$$\begin{array}{ll} \dot{V} & =-(I_{Ca}+I_{Kdr}+I_{leak}+I_{K1}+I_{K2})/C_{m},\\ \dot{n} & =(n_{\infty }(V)-n)/\tau_{n}(V),\\ {\dot{s}}_{1} & =(s_{1\infty }(V)-s_{1})/\tau_{s_{1}},\\ {\dot{s}}_{2} & =(s_{2\infty }(V)-s_{2})/\tau_{s_{2}}, \end{array}$$ (19)

where we refer to [46] and the SI of the present article for details on the various ionic currents and gating functions, as well as for the initial parameter set, taken to be that of Fig 3 from [46], reproduced in Fig 13 panels (a1)-(a2). The model was reported to exhibit square-wave bursting dynamics, and also it was noted to sustain a more complicated oscillations with “small wiggles” in the quiescent phase. To explain this phenomenon, a slow-fast dissection was performed whereby the fast 3D subsystem was obtained by freezing the slowest of the 2 slow variables, *s*_2_ [46]. The conclusion of the authors’ analysis was that the small oscillations occurring during the quiescent phase can be interpreted as the result of a slow passage through a Hopf bifurcation taking place in this 3D fast subsystem. However, it turns out that this explanation is valid to only a certain extent and fails to explain parsimoniously the complete phenomenon. As alluded by our proposed classification extension, both slow variables of the system suspiciously play a role in shaping these small wiggles. Following our proposed decomposition, we reveal the presence of a folded node in the corresponding 2D slow subsystem, which further elucidates the mechanisms that controls the number of small wiggles depending on system parameters; see Fig 13(a1) and 13(a2). What is more, when modifying the kinetics of both slow currents, making the timescales of the slow variables closer to each other than in the original parameter set, one can exhibit a different parameter set in which the folded-node scenario still explains the presence of small wiggles during the quiescent phase of the bursting pattern whereas the Hopf bifurcation is unable to do so. Indeed, in this scenario, the Hopf point of the 3D fast subsystem obtained by considering *s*_2_ as the only slow variable in the model is now located outside of the region of subthreshold oscillations. In conclusion, the folded-node scenario is more robust and parsimonious at explaining this bursting pattern; see Fig 13(b1) and 13(b2).

## Conclusions

The mathematical classification of bursting patterns was initiated with seminal papers published in the mid-1980s with 3 proposed classes of bursting oscillations [6,19,41]. The key idea of comparing the fast subsystem’s bifurcation diagram and the full systems’ dynamics may seem natural with hindsight, but in fact, it was a genuine breakthrough, which shaped the way bursting oscillations have been modeled and dissected ever since.

The present review details these footsteps, as well as those of the subsequent contributors on this topic [7–9], hence it was important to gather these results since they form one pillar of mathematical neuroscience and computational biology, but also have impact in other fields. We then take a step forward by proposing an extension of the classification scheme, which allows to cover more types of bursting systems, in particular fold-initiated bursters with 2 slow variables, namely folded-node bursters. The extended bursting classification crucially focuses on the dynamics during the silent phase where the termination of the trajectory profile is not just a simple rise over the fold of the critical manifold but can involve subthreshold oscillations.

We emphasize the importance of the slow flow (*ε* = 0) in slow-fast systems with (at least) 2 slow variables, which was somehow previously overlooked in the context of bursting. In such two-slow-variable bursting systems, the silent phase termination is due to the presence of folded node. This scenario is known to give rise to canard solutions that organize, upon parameter variation but also transiently, upon change of initial conditions, the number of subthreshold oscillations. This slow cycle-adding phenomenon is indeed entirely due to canards and it controls the profile of the underlying bursting oscillations. Importantly, it does so in a robust manner in the sense that such bursting patterns with subthreshold oscillations exist over order-one ranges of parameter values. Parabolic bursters have 2 slow variables as well; however, their slow flow possesses a folded saddle and not a folded node [45]. In this context, it will be interesting to study further the transition between some folded-node bursters like folded-node homoclinic bursters and parabolic bursters with a multiparameter unfolding of the transition in both slow and fast singular limits, where folded-saddle-node singularities and saddle-node homoclinic bifurcations could play key roles, respectively. In summary, we have reviewed the state-of-the-art bursting classification and enhanced it so as to take into account both slow and fast subsystems. Indeed, the slow singular limit, where the fast variables are slaved to the slow ones and the dynamics is constrained to the critical manifold, had not been taken into account in previous classification schemes. This enables to capture a larger class of complex oscillations.

Where do we go from here? Following this initial framework for folded-node bursting, it will be important to develop this approach in the context of biophysical excitable cell models with more than one slow process. To this extent, an interesting question for follow-up work is to rethink folded-node bursting dynamics from a biophysical modeling viewpoint. In all our idealized models of folded-node bursting, we have added feedback terms in the second slow differential equation with both positive and negative coefficients, which tends to indicate that both positive and negative feedback loops are useful to produce the desired output behavior.

In this context, we highlight 2 interesting aspects associated with the experimental time series that we attempted to model with our idealized folded-node bursting model reproduced in Figs 3 and 8. First, the subthreshold oscillations appear to be following the excitability threshold, which may be harder to obtain in a 3D model, even though some elliptic bursting models—e.g., FitzHugh–Rinzel, Morris–Lecar as well as some MMO models—could potentially reproduce this aspect. Note that our example of folded-node bursting has 3 time scales; this was done for convenience in the construction and may not be absolutely necessary.

Second, the burst phase is located on a plateau (in terms of neuronal membrane potential values) compared to the quiescent phase, which is reminiscent of a square-wave type bursting. Indeed, our idealized folded-node bursting model reproduces quite well these data and, in fact, it can effectively be designated as a folded-node homoclinic bursting model. Three-dimensional elliptic bursting models, or MMO models, would not be able to capture this aspect.

One interesting possibility to find biophysical models with folded-node bursting dynamics is perhaps via existing models of thalamic bursting, or alternatively to extend these models to explain the observational data published in [31]. When it comes to biologically plausible models, where the timescale separation may not be explicit or in standard form, the recent theoretical work by Wechselberger and colleagues on extending slow-fast theory to systems in so-called *nonstandard* form (see, e.g., [86]) may allow to derive new mechanisms and new bursting patterns.

In terms of application to neural dynamics, it is legitimate to ask about neural coding [87–89] and the implications of folded-node dynamics within a bursting regime. There, one would want to compare spike-adding to folded-node cycle-adding. The cycle adding can quantize the slow phase duration, which might have significant effect on silent phase (and therefore on active phase) durations.

On the other hand, spike-adding has less impact on macroscopic timing and less impact if a spike is added to a burst of several, say, 6 or more, spikes. A single spike added in a 2- to 4-spike burst might have coding contributions (synaptic transmission) but less so if there are already more than 6 spikes in a burst. These questions go beyond the scope of the present paper but are certainly of direct interest for follow-up work.

The question of noise is also a natural one to consider. If small to moderate noise is added to a folded-node bursting systems, it will likely not affect significantly the burst phase. However, it is expected that the phase of spiking oscillations during the burst will be affected, but not the qualitative dynamics. Folded-node dynamics is known to be robust to noise; its time course is parametrically robust and noise tolerant. The canard phenomenon accounts for subtle dynamic features like cycle-adding; however, the subthreshold oscillations near a folded node are robust. The noise will affect these subthreshold oscillations by modifying the rotation sector in which the trajectory falls into from one passage to the the next; however, the oscillations will remain.

To quantify this variability of the sector of a folded-node burster with noise, one could use results by Berglund and colleagues [90]. However, here as well the qualitative dynamics and the key role of the slow subsystem and its folded node will remain. A rigorous understanding of the impact of noise on a folded-node bursting model is certainly an interesting question that goes beyond the scope of the present work.

Finally, the question of bursting dynamics with at least 2 slow variables and more than 2 timescales is also of interest and related to the present work. As aforementioned, in the limit of folded-saddle-node singularities, small subthreshold oscillations will remain and increase in number and shape. In the context of slow-fast systems with 2 slow variables, this scenario is well known to be akin to 3-timescale dynamics [91]. The associated bifurcation structure is already involved in the 3D setup, with involvement of adding organizing centers such as singular Hopf bifurcation points [92]. Thus, the folded-saddle-node bursting profiles will be more rich and complex to fully describe than the folded-node bursting cases presented herein. Yet, the underlying robust mechanism that gives a bursting pattern and requires the analysis of both slow and fast subsystem will be similar as the one proposed in the present work. A full analysis of this limiting case is a very interesting and natural question for future work. Besides, bursting systems with more than 2 timescales have recently gained further interest in link with canards [76,93–95], where the additional timescales bring more structure to the system and allow for further slow-fast analysis. Such approaches would certainly shed further light onto folded-node bursting dynamics as presented here, and we regard it as a natural and interesting question for future work.

## Supporting information

S1 Text More details about the models, parameter values, and technical terms. (PDF)

## References

[1] Hedges ES, Myers JE. The Problem of Physico-chemical Periodicity. Arnold, London; 1926.

[2] Appleton EV, van der Pol B. XVI. On a type of oscillation-hysteresis in a simple triode generator. Lond Edinb Dublin Philos Mag J Sci. 1922;43(253):177–193.

[3] Van der Pol B. LXXXVIII. On “relaxation-oscillations”. Lond Edinb Dublin Philos Mag J Sci. 1926;2(11):978–92.

[4] Van Der Pol B, Van Der Mark J. LXXII. The heartbeat considered as a relaxation oscillation, and an electrical model of the heart. Lond Edinb Dublin Philos Mag J Sci. 1928;6(38):763–775.

[5] Coombes S, Bressloff PC. Bursting: the genesis of rhythm in the nervous system. World Scientific; 2005.

[6] Rinzel J. A Formal Classification of Bursting Mechanisms in Excitable Systems. In: International Congress of Mathematicians, Berkeley, California, USA, August 3–11 vol II. American Mathematical Society. 1986;1987:1578–93.

[7] Izhikevich EM. Neural excitability, spiking and bursting. Int J Bifurc Chaos. 2000;10(06):1171–266.

[8] Bertram R, Butte MJ, Kiemel T, Sherman A. Topological and phenomenological classification of bursting oscillations. Bull Math Biol. 1995;57(3):413–39. doi: 10.1007/BF02460633 7728115

[9] Golubitsky M, Josic K, Kaper TJ. An unfolding theory approach to bursting in fast-slow systems. In: Broer HW, Krauskopf B, Vegter G, editors. Global analysis of dynamical systems. IOP Publishing; 2001. p. 277–308.

[10] Desroches M, Guckenheimer J, Krauskopf B, Kuehn C, Osinga HM, Wechselberger M. Mixed-mode oscillations with multiple time scales. SIAM Rev. 2012;54(2):211–88.

[11] Desroches M, Guillamon A, Ponce E, Prohens R, Rodrigues S, Teruel AE. Canards, folded nodes, and mixed-mode oscillations in piecewise-linear slow-fast systems. SIAM Rev. 2016;58(4):653–91.

[12] Holmes WR, Park J, Levchenko A, Edelstein-Keshet L. A mathematical model coupling polarity signaling to cell adhesion explains diverse cell migration patterns. PLoS Comput Biol. 2017;13(5):e1005524. doi: 10.1371/journal.pcbi.1005524 28472054 PMC5436877

[13] Kimrey J, Vo T, Bertram R. Canard analysis reveals why a large Ca2+ window current promotes early afterdepolarizations in cardiac myocytes. PLoS Comput Biol. 2020;16(11):e1008341. doi: 10.1371/journal.pcbi.1008341 33147207 PMC7641359

[14] McKenna JP, Dhumpa R, Mukhitov N, Roper MG, Bertram R. Glucose oscillations can activate an endogenous oscillator in pancreatic islets. PLoS Comput Biol. 2016;12(10):e1005143. doi: 10.1371/journal.pcbi.1005143 27788129 PMC5082885

[15] Rho YA, Prescott SA. Identification of molecular pathologies sufficient to cause neuropathic excitability in primary somatosensory afferents using dynamical systems theory. PLoS Comput Biol. 2012;8(5):e1002524. doi: 10.1371/journal.pcbi.1002524 22654655 PMC3359967

[16] Ullah G, Wei Y, Dahlem MA, Wechselberger M, Schiff SJ. The role of cell volume in the dynamics of seizure, spreading depression, and anoxic depolarization. PLoS Comput Biol. 2015;11(8):e1004414.po b doi: 10.1371/journal.pcbi.1004414 26273829 PMC4537206

[17] Yu N, Morris CE, Joós B, Longtin A. Spontaneous excitation patterns computed for axons with injury-like impairments of sodium channels and Na/K pumps. PLoS Comput Biol. 2012;8(9):e1002664. doi: 10.1371/journal.pcbi.1002664 23028273 PMC3441427

[18] Moehlis J. Canards for a reduction of the Hodgkin-Huxley equations. J Math Biol. 2006;52(2):141–53. doi: 10.1007/s00285-005-0347-1 16195925

[19] Rinzel J. Bursting oscillations in an excitable membrane model. In: Sleeman BD, Jarvis RJ, editors. Ordinary and partial differential equations (Proceedings of the Eighth Conference held at Dundee, Scotland, June 25–29, 1984). vol. 1511 of Lecture Notes in Mathematics. Springer; 1985. p. 304–316.

[20] Iglesias C, Meunier C, Manuel M, Timofeeva Y, Delestrée N, Zytnicki D. Mixed mode oscillations in mouse spinal motoneurons arise from a low excitability state. J Neurosci. 2011;31(15):5829–40. doi: 10.1523/JNEUROSCI.6363-10.2011 21490224 PMC6622841

[21] Desroches M, Krupa M, Rodrigues S. Inflection, canards and excitability threshold in neuronal models. J Math Biol. 2013;67(4):989–1017. doi: 10.1007/s00285-012-0576-z 22945512

[22] Wechselberger M, Mitry J, Rinzel J. Canard theory and excitability. In: Kloeden P, Pötzsche C, editors. Nonautonomous dynamical systems in the life sciences. vol. 2102 of Lecture Notes in Mathematics. Springer; 2013. p. 89–132.

[23] Benoît E, Callot JL, Diener F, Diener M. Chasse au canard. Collect Math. 1981;32(1–2):37–119.

[24] FitzHugh R. Impulses and physiological states in theoretical models of nerve membrane. Biophys J. 1961;1(6):445–66. doi: 10.1016/s0006-3495(61)86902-6 19431309 PMC1366333

[25] Nagumo J, Arimoto S, Yoshizawa S. An active pulse transmission line simulating nerve axon. Proc IRE. 1962;50(10):2061–70.

[26] Newcomb JM. CeN Inhibits SMPjpg; 2008. http://neuronbank.org/wiki/index.php/File:CeN_Inhibits_SMP.jpg.

[27] Riz M, Braun M, Pedersen MG. Mathematical modeling of heterogeneous electrophysiological responses in human *β*-cells. PLoS Comput Biol. 2014;10(1):e1003389. doi: 10.1371/journal.pcbi.1003389 24391482 PMC3879095

[28] Jian Z, Xing JL, Yang GS, Hu SJ. A novel bursting mechanism of type a neurons in injured dorsal root ganglia. Neurosignals. 2004;13(3):150–6. doi: 10.1159/000076569 15067203

[29] Tabak J, Tomaiuolo M, Gonzalez-Iglesias AE, Milescu LS, Bertram R. Fast-activating voltage-and calcium-dependent potassium (BK) conductance promotes bursting in pituitary cells: a dynamic clamp study. J Neurosci. 2011;31(46):16855–63. doi: 10.1523/JNEUROSCI.3235-11.2011 22090511 PMC3252383

[30] Rinzel J, Ermentrout GB. Analysis of neural excitability and oscillations. Methods in neuronal modeling. vol. 2. MIT press Cambridge, MA; 1998. p. 251–292.

[31] Roy JP, Clercq M, Steriade M, Deschênes M. Electrophysiology of neurons of lateral thalamic nuclei in cat: mechanisms of long-lasting hyperpolarizations. J Neurophysiol. 1984;51(6):1220–35. doi: 10.1152/jn.1984.51.6.1220 6429291

[32] Fenichel N. Geometric singular perturbation theory for ordinary differential equations. J Differ Equ. 1979;31(1):53–98.

[33] Hek G. Geometric singular perturbation theory in biological practice. J Math Biol. 2010;60(3):347–86. doi: 10.1007/s00285-009-0266-7 19347340

[34] Teka W, Stockton D, Santamaria F. Power-law dynamics of membrane conductances increase spiking diversity in a Hodgkin-Huxley model. PLoS Comput Biol. 2016;12(3):e1004776. doi: 10.1371/journal.pcbi.1004776 26937967 PMC4777484

[35] Desroches M, Kaper TJ, Krupa M. Mixed-mode bursting oscillations: Dynamics created by a slow passage through spike-adding canard explosion in a square-wave burster. Chaos: An Interdisciplinary J Nonlinear Sci. 2013;23(4):046106. 10.1063/1.4827026 24387585

[36] Köksal Ersöz E, Desroches M, Guillamon A, Rinzel J, Tabak J. Canard-induced complex oscillations in an excitatory network. J Math Biol. 2020;80(7):2075–107. doi: 10.1007/s00285-020-01490-1 32266428

[37] Teka W, Tsaneva-Atanasova K, Bertram R, Tabak J. From plateau to pseudo-plateau bursting: Making the transition. Bull Math Biol. 2011;73(6):1292–311. doi: 10.1007/s11538-010-9559-7 20658200 PMC3152987

[38] Bertram R, Tabak J, Teka W, Vo T, Wechselberger M. Geometric Singular Perturbation Analysis of Bursting Oscillations in Pituitary Cells. In: Mathematical analysis of complex cellular activity. Springer; 2015. p. 1–52.

[39] Vo T, Bertram R, Wechselberger M. Multiple geometric viewpoints of mixed mode dynamics associated with pseudo-plateau bursting. SIAM J Appl Dyn Syst. 2013;12(2):789–830.

[40] Hindmarsh JL, Rose R. A model of neuronal bursting using three coupled first order differential equations. Proc R Soc Lond B Biol Sci. 1984;221(1222):87–102. doi: 10.1098/rspb.1984.0024 6144106

[41] Rinzel J. A formal classification of bursting mechanisms in excitable systems. In: Teramoto E, Yumaguti M, editors. Mathematical topics in population biology, morphogenesis and neurosciences (Proceedings of an International Symposium held in Kyoto, November 10–15, 1985). vol. 71 of Lecture Notes in Biomathematics. Springer; 1987. p. 267–281.

[42] Plant RE. Bifurcation and resonance in a model for bursting nerve cells. J Math Biol. 1981;11(1):15–32. doi: 10.1007/BF00275821 7252375

[43] Farjami S, Alexander RP, Bowie D, Khadra A. Bursting in cerebellar stellate cells induced by pharmacological agents: Non-sequential spike adding. PLoS Comput Biol. 2020;16(12):e1008463. doi: 10.1371/journal.pcbi.1008463 33315892 PMC7769625

[44] Kramer MA, Traub RD, Kopell NJ. New dynamics in cerebellar purkinje cells: torus canards. Phys Rev Lett. 2008;101(6):068103. doi: 10.1103/PhysRevLett.101.068103 18764509 PMC2662447

[45] Desroches M, Krupa M, Rodrigues S. Spike-adding in parabolic bursters: The role of folded-saddle canards. Physica D: Nonlinear Phenomena. 2016;331:58–70.

[46] Bertram R, Rhoads J, Cimbora WP. A phantom bursting mechanism for episodic bursting. Bull Math Biol. 2008;70(7):1979. doi: 10.1007/s11538-008-9335-0 18648884

[47] Yildirim V, Bertram R. Calcium oscillation frequency-sensitive gene regulation and homeostatic compensation in pancreatic *β*-Cells. Bull Math Biol. 2017;79(6):1295–1324. doi: 10.1007/s11538-017-0286-1 28497293

[48] Belykh I, De Lange E, Hasler M. Synchronization of bursting neurons: What matters in the network topology. Phys Rev Lett. 2005;94(18):188101. doi: 10.1103/PhysRevLett.94.188101 15904412

[49] Venugopal S, Seki S, Terman DH, Pantazis A, Olcese R, Wiedau-Pazos M, et al. Resurgent Na+ current offers noise modulation in bursting neurons. PLoS Comput Biol. 2019;15(6):e1007154. doi: 10.1371/journal.pcbi.1007154 31226124 PMC6608983

[50] Kepecs A, Wang XJ. Analysis of complex bursting in cortical pyramidal neuron models. Neurocomputing. 2000;32:181–7.

[51] Izhikevich EM. Dynamical Systems in Neuroscience: the Geometry of Excitability and Bursting. Computational neuroscience. Cambridge, Mass: MIT Press; 2007.

[52] Köksal Ersöz E, Modolo J, Bartolomei F, Wendling F. Neural mass modeling of slow-fast dynamics of seizure initiation and abortion. PLoS Comput Biol. 2020;16(11):e1008430. doi: 10.1371/journal.pcbi.1008430 33166277 PMC7676664

[53] Hübel N, Hosseini-Zare MS, Žiburkus J, Ullah G. The role of glutamate in neuronal ion homeostasis: A case study of spreading depolarization. PLoS Comput Biol. 2017;13(10):e1005804. doi: 10.1371/journal.pcbi.1005804 29023523 PMC5655358

[54] Saggio ML, Spiegler A, Bernard C, Jirsa VK. Fast–Slow Bursters in the Unfolding of a High Codimension Singularity and the Ultra-slow Transitions of Classes. J Math Neurosci. 2017;7(1):7. doi: 10.1186/s13408-017-0050-8 28744735 PMC5526832

[55] Rubin J, Krauskopf B, Osinga H. Natural extension of fast-slow decomposition for dynamical systems. Phys Rev E. 2018;97(1):012215. doi: 10.1103/PhysRevE.97.012215 29448375

[56] Desroches M, Rinzel J, Rodrigues S. Towards a new classification of bursting patterns: review & extensions. arXiv eprint 2020;(2001.09625).

[57] Desroches M, Kowalczyk P, Rodrigues S. Spike-adding and reset-induced canard cycles in adaptive integrate and fire models. Nonlinear Dynamics. 2021;104(3):2451–70.

[58] Fardet T, Levina A. Simple models including energy and spike constraints reproduce complex activity patterns and metabolic disruptions. PLoS Comput Biol. 2020;16(12):e1008503. doi: 10.1371/journal.pcbi.1008503 33347433 PMC7785241

[59] Górski T, Depannemaecker D, Destexhe A. Conductance-based Adaptive Exponential integrate-and-fire model. Neural Comput. 2021;33(1):41–66. doi: 10.1162/neco_a_01342 33253029

[60] Rubin JE, Signerska-Rynkowska J, Touboul J, Vidal A. Wild oscillations in a nonlinear neuron model with resets:(I) Bursting, spike adding and chaos. Discrete Continuous Dyn Syst Ser B. 2016;22(10):3967–4002.

[61] Smith GD, Cox CL, Sherman SM, Rinzel J. Fourier analysis of sinusoidally driven thalamocortical relay neurons and a minimal integrate-and-fire-or-burst model. J Neurophysiol. 2000;83(1):588–610. doi: 10.1152/jn.2000.83.1.588 10634897

[62] Izhikevich EM, Hoppensteadt F. Classification of bursting mappings. Int J Bifurc Chaos. 2004;14(11):3847–54.

[63] Eckhaus W. Relaxation oscillations including a standard chase on French ducks. In: Verhulst F, editor. Asymptotic Analysis II. vol. 985 of Lecture Notes in Mathematics. Springer; 1983. p. 449–497.

[64] Krupa M, Szmolyan P. Relaxation oscillation and canard explosion. J Differ Equ. 2001;174(2):312–68.

[65] Mishchenko EF, Kolesov S, Kolesov YA, Rozov NK. Asymptotic methods in singularly perturbed systems. Consultants Bureau; 1994.

[66] Benoît E. Canards et enlacements. Publications Mathématiques de l’Institut des Hautes Études Scientifiques. 1990;72(1):63–91.

[67] Wechselberger M. Existence and Bifurcation of Canards in $R^{3}$ in the Case of a Folded Node. SIAM J Appl Dyn Syst. 2005;4(1):101–39.

[68] De Maesschalck P, Wechselberger M. Neural excitability and singular bifurcations. J Math Neurosci. 2015;5(1):16. doi: 10.1186/s13408-015-0029-2 26246435 PMC4526515

[69] Terman D. Chaotic spikes arising from a model of bursting in excitable membranes. SIAM J Appl Math. 1991;51(5):1418–50.

[70] Guckenheimer J, Kuehn C. Computing slow manifolds of saddle type. SIAM J Appl Dyn Syst. 2009;8(3):854–79.

[71] Nowacki J, Osinga HM, Tsaneva-Atanasova K. Dynamical systems analysis of spike-adding mechanisms in transient bursts. J Math Neurosci. 2012;2(1):7. doi: 10.1186/2190-8567-2-7 22655748 PMC3497719

[72] Tsaneva-Atanasova K, Osinga HM, Rieß T, Sherman A. Full system bifurcation analysis of endocrine bursting models. J Theor Biol. 2010;264(4):1133–46. doi: 10.1016/j.jtbi.2010.03.030 20307553 PMC3128456

[73] Osinga HM, Sherman A, Tsaneva-Atanasova K. Cross-currents between biology and mathematics: The codimension of pseudo-plateau bursting. Discrete Continuous Dyn Syst Ser A. 2012;32(8):2853. doi: 10.3934/dcds.2012.32.2853 22984340 PMC3439852

[74] Dumortier F, Roussarie R, Sotomayor J, Zoladek H. Bifurcations of planar vector fields: nilpotent singularities and Abelian integrals. vol. 1480 of Lecture Notes in Mathematics. Springer-Verlag; 1991.

[75] Morris C, Lecar H. Voltage oscillations in the barnacle giant muscle fiber. Biophys J. 1981;35(1):193–213. doi: 10.1016/S0006-3495(81)84782-0 7260316 PMC1327511

[76] Desroches M, Kirk V. Spike-adding in a canonical three-time-scale model: superslow explosion and folded-saddle canards. SIAM J Appl Dyn Syst. 2018;17(3):1989–2017.

[77] Mitry J, Wechselberger M. Folded saddles and faux canards. SIAM J Appl Dyn Syst. 2017;16(1):546–96.

[78] Izhikevich EM. Synchronization of elliptic bursters. SIAM Rev. 2001;43(2):315–44.

[79] Benes GN, Barry AM, Kaper TJ, Kramer MA, Burke J. An elementary model of torus canards. Chaos: An Interdisciplinary J Nonlinear Sci. 2011;21(2):023131. doi: 10.1063/1.3592798 21721773

[80] Burke J, Desroches M, Barry AM, Kaper TJ, Kramer MA. A showcase of torus canards in neuronal bursters. J Math Neurosci. 2012;2(1):3. doi: 10.1186/2190-8567-2-3 22657918 PMC3496470

[81] Vo T. Generic torus canards. Physica D: Nonlinear Phenomena 2017;356–357:37–64.

[82] Vo T, Kramer MA, Kaper TJ. Amplitude-Modulated Bursting: A Novel Class of Bursting Rhythms. Phys Rev Lett. 2016;117(26):268101. doi: 10.1103/PhysRevLett.117.268101 28059538

[83] Han X, Wei M, Bi Q, Kurths J. Obtaining amplitude-modulated bursting by multiple-frequency slow parametric modulation. Phys Rev E. 2018;97(1):012202. doi: 10.1103/PhysRevE.97.012202 29448416

[84] Roberts KL, Rubin JE, Wechselberger M. Averaging, folded singularities, and torus canards: explaining transitions between bursting and spiking in a coupled neuron model. SIAM J Appl Dyn Syst. 2015;14(4):1808–44.

[85] Roberts KL. Geometric Singular Perturbation Theory and Averaging: Analysing Torus Canards in Neural Models. School of Mathematics and Statistics. University of Sydney; 2018.

[86] Wechselberger M. Geometric singular perturbation theory beyond the standard form. Springer; 2020.

[87] Izhikevich EM. Resonance and selective communication via bursts in neurons having subthreshold oscillations. Biosystems. 2002;67(1–3):95–102. doi: 10.1016/s0303-2647(02)00067-9 12459288

[88] Izhikevich EM, Desai NS, Walcott EC, Hoppensteadt FC. Bursts as a unit of neural information: selective communication via resonance. Trends Neurosci. 2003;26(3):161–7. doi: 10.1016/S0166-2236(03)00034-1 12591219

[89] Zeldenrust F, Chameau P, Wadman WJ. Spike and burst coding in thalamocortical relay cells. PLoS Comput Biol. 2018;14(2):e1005960. doi: 10.1371/journal.pcbi.1005960 29432418 PMC5834212

[90] Berglund N, Gentz B, Kuehn C. Hunting French ducks in a noisy environment. J Differ Equ. 2012;252(9):4786–841.

[91] Krupa M, Wechselberger M. Local analysis near a folded saddle-node singularity. J Differ Equ. 2010;248(12):2841–88.

[92] Guckenheimer J. Singular Hopf bifurcation in systems with two slow variables. SIAM J Appl Dyn Syst. 2008;7(4):1355–77.

[93] Krupa M, Vidal A, Desroches M, Clément F. Mixed-mode oscillations in a multiple time scale phantom bursting system. SIAM J Appl Dyn Syst. 2012;11(4):1458–98.

[94] Letson B, Rubin JE, Vo T. Analysis of interacting local oscillation mechanisms in three-timescale systems. SIAM J Appl Math. 2017;77(3):1020–46.

[95] Nan P, Wang Y, Kirk V, Rubin JE. Understanding and distinguishing three-time-scale oscillations: Case study in a coupled Morris–Lecar system. SIAM J Appl Dyn Syst. 2015;14(3):1518–1557.

