# Supplementary material for: Classification of bursting patterns: A tale of two ducks
Source: PLoS Comput Biol. 2022 Feb 24;18(2):e1009752. doi: 10.1371/journal.pcbi.1009752 (PMC8870467; doi:10.1371/journal.pcbi.1009752)
Supplement: S1 Text — (PDF) [file pcbi.1009752.s001.pdf]

Supporting Information for the article entitled:
Classification of bursting patterns: A tale of two ducks

Mathieu Desroches[1,2*], John Rinzel[3,4], Serafim Rodrigues[2,5*]

1 MathNeuro Team, Inria Sophia Antipolis Méditerranée Research Centre, Sophia Antipolis, France
2 MCEN Team, Basque Centre for Applied Mathematics (BCAM), Bilbao, Bizkaia, Spain
3 Center for Neural Science, New York University, New York, NY, USA
4 Courant Institute for Mathematical Sciences, New York University, New York, NY, USA
5 Ikerbasque, The Basque Science Foundation

* mathieu.desroches@inria.fr, srodrigues@bcamath.org

Abstract

In this supplementary document, we provide all parameter values and equations for all figures of the main article. We also provide a glossary of symbols used in Figs 1 and 4–13.

Equations and parameter values for Fig 1.

Spiking example: FitzHugh-Nagumo model

Equations:

$$\begin{aligned} V' &= V - V^3/3 - w + I \\ w' &= \varepsilon(v + a - bw) \end{aligned}$$

Parameter values:

$\varepsilon = 0.01$, $a = 0.7$, $b = 0.8$ and: **(a)** I=-0.43, **(b)** I=0.87, **(c)** $I \sim I_T = 0.29572901011$. In panel (a), to observe the excitable character of the stable equilibrium (located at $(-1.418, -0.89755)$), we take an initial condition at the equilibrium and apply a step of current (from the baseline $I_0 = -0.43$) at time $t = 10$ and of duration $\Delta t = 0.1$. With a step amplitude of $I_1 = 9.354$, we obtain a trajectory (red) that remains below threshold and converges back to the stable equilibrium; with a step amplitude of $I_1 = 9.355$, we obtain a trajectory (purple) that crosses the threshold, fires an action potential and then converges back to the stable equilibrium (rest state).

Equations and parameter values for Fig 4.

Panels (a1)-(b1): Square-wave example

Equations: Hindmarsh-Rose model

$$\begin{aligned} V' &= y - aV^3 + bV^2 - I_{\text{slow}} + I \\ w' &= c - dV^2 - w \\ I'_{\text{slow}} &= \varepsilon(s(V - x_1) - I_{\text{slow}}) \end{aligned}$$

Parameter values:

$a = 1$, $b = 3$, $I = 2.15$, $c = 1$, $d = 5$, $\varepsilon = 0.0003$, $s = 4$, $x_1 = -1.618$.

Panels (a2)-(b2): Elliptic bursting example

Equations: FitzHugh-Rinzel model

$$\begin{aligned} V' &= V - V^3/3 - w + I_{\text{slow}} + I \\ w' &= \phi(V + a - bw) \\ I'_{\text{slow}} &= \varepsilon(-V + c - dI_{\text{slow}}) \end{aligned}$$

Parameter values:

$I = 0.31$, $\phi = 0.08$, $a = 0.7$, $b = 0.8$, $\varepsilon = 0.0001$, $c = -0.775$, $d = 1$.

Panels (c1)-(c2): Parabolic bursting example

Equations: Plant model

$$\begin{aligned} C_m V' &= -\bar{g}_I m_\infty^3(V) h\,(V - V_I) - \bar{g}_T x\,(V - V_I) - \bar{g}_K n^4\,(V - V_K) + \bar{g}_{KCa} \frac{Ca}{0.5 + Ca}\,(V - V_K) - \bar{g}_L\,(V - V_L) \\ h' &= \frac{h_\infty(V) - h}{\tau_h(V)} \\ n' &= \frac{h_\infty(V) - h}{\tau_h(V)} \\ x' &= \frac{h_\infty(V) - h}{\tau_h(V)} \\ Ca' &= \varepsilon(-K_c x(V - V_{Ca}) - Ca) \end{aligned}$$

The steady-state activation and inactivation functions are given by

$$\begin{aligned} w_\infty(V) &= \frac{\alpha_w(V)}{\alpha_w(V) + \beta_w(V)} \quad \text{for } w = m,\ h,\ n \\ \tau_w(V) &= \frac{12.5}{\alpha_w(V) + \beta_w(V)} \quad \text{for } w = h,\ n \end{aligned}$$

and

$$x_\infty(V) = \frac{1}{\exp(-0.3(V + 40) + 1)},$$

where

$$\begin{aligned}
\alpha_m(V) &= 0.1\frac{50 - V_s}{\exp\left(\left(50 - V_s\right)/10\right) - 1} \\
\beta_m(V) &= 4\exp\left(\left(25 - V_s\right)/18\right) \\
\alpha_h(V) &= 0.07\exp\left(\left(25 - V_s\right)/20\right) \\
\beta_h(V) &= \frac{1}{\exp\left(\left(55 - V_s\right)/10\right) + 1} \\
\alpha_n(V) &= 0.01\frac{55 - V_s}{\exp\left(\left(55 - V_s\right)/10\right) - 1} \\
\beta_n(V) &= 0.125\exp\left(\left(45 - V_s\right)/80\right),
\end{aligned}$$

and

$$V_s = \frac{127}{105}V + \frac{8265}{105}.$$

Parameter values (units):

$C_m = 1$ (μF/cm^2), $\bar{g}_I = 4$ (mmho/cm^2), $\bar{g}_T = 0.01$ (mmho/cm^2), $\bar{g}_K = 0.3$ (mmho/cm^2), $\bar{g}_{KCa} = 0.03$ (mmho/cm^2), $\bar{g}_L = 0.003$ (mmho/cm^2), $V_I = 30$ (mV), $V_K = -75$ (mV), $V_L = -40$ (mV), $V_{Ca} = 140$ (mV), $\varepsilon = 0.00015$ (ms^{-1}), $K_c = 0.00425$ (mV^{-1}), $\tau_x = 9400$ (ms).

Glossary of symbols used in Figs 1 and 4–13.

Table A. Glossary of technical terms used in the figures.

Symbol	Name & description	Figures
I_T	Value of the applied current at which the threshold to tonic firing is crossed in the FitzHugh-Nagumo model	1
LP •	Saddle-node bifurcation of equilibria (*fold* or *Limit Point*)	4(a1),5(a),7(d1) 7(b2)-(c2),8(a),9(a),10(a)
Ho ⋆	Saddle-homoclinic bifurcation	4(a1),5(a),7(d1)-(b2)-(c2) 8(a)
HB •	Hopf bifurcation	4(a1)-(a2),5(a), 7(b2)-(c2),8(a)-(b) 9(a)-(b),10(b),13(a1)-(b1)
SNP •	Saddle-node bifurcation of limit cycles (*cyclic fold*)	4(a2),10(a)
S^0	Critical manifold (i.e. locus of points in phase space at which the fast components of the vector field vanish)	4(a3),8(d),9(d),10(d) 13(a1)-(b1)
S^0_a	Attracting part of the critical manifold	6(b),7(b1)-(d1)-(b2) 7(c2)-(c3)-(d3)
S^0_r	Repelling part of the critical manifold	6(b),7(b1)-(d1)-(b2) 7(c2)-(b3)-(c3)
$\mathcal{F}$, $\mathcal{F}^{\pm}$	fold curve of the critical manifold (lower of upper one)	4(a3),6(b),7(b1)-(b3)-(c3) 8(d),9(d),10(d),13(a1), 13(b1)
fn •	Folded node	6(b),7(b1)-(c1)-(b3) 7(c3)-(d3),8(d),9(d),10(d) 13(a1)-(b1)
S^ε_a	Attracting slow manifold (i.e. perturbation of an attracting part of the critical manifold)	6(b),7(c1)
S^ε_r	Repelling slow manifold (i.e. perturbation of a repelling part of the critical manifold)	6(b),7(c1)
— LP	Curve of saddle-node bifurcation points in 2 parameters	7(d2)-(d3)
— Ho	Curve of saddle-homoclinic bifurcation points in 2 parameters	7(b1)-(d2)
— HB	Curve of Hopf bifurcation points in 2 parameters	7(d2)
BT •	Bodganov-Takens bifurcation point in a 2-parameter plane	7(d2)

Equations and parameter values for Fig 8.

Panels (a)-(b): fold-homoclinic (square-wave) bursting example

Equations:

$$
\begin{aligned}
x' &= (y - x^3 + 3x^2 + az)/c \\
y' &= 1 - 5x^2 - y \\
z' &= \varepsilon(\alpha x + \gamma\beta - \delta z)
\end{aligned}
$$

Parameter values:

$a = 1$, $c = 1$, $\varepsilon = 0.002$, $\alpha = 0.3$, $\gamma = 1$, $\delta = 1.2$.

Panels (c)-(d): folded-node/homoclinic bursting example

Equations:

$$
\begin{aligned}
x' &= (y - x^3 + 3x^2 + az)/c \\
y' &= 1 - 5x^2 - y \\
z' &= \varepsilon(\alpha x + \gamma\beta - \delta z) \\
\beta' &= \varepsilon\big(\mu - \gamma_y(y - y_f)^2 - \gamma_\beta(\beta - \beta_f)^2\big)
\end{aligned}
$$

Parameter values:

$a = 1$, $c = 1$, $\varepsilon = 0.002$, $\alpha = 0.3$, $\gamma = 1$, $\delta = 1.2$, $\mu = 0.033$, $\gamma_y = 0.0005$ and $\gamma_\beta = -0.008$.

Equations and parameter values for Figure 9.

Panels (a)-(b): fold-Hopf bursting example

Equations:

$$
\begin{aligned}
x' &= (y - x^3 + 3x^2 + az)/c \\
y' &= 1 - 5x^2 - y \\
z' &= \varepsilon(\alpha x + \gamma\beta - \delta z)
\end{aligned}
$$

Parameter values:

$a = 1$, $c = 2$, $\varepsilon = 0.004$, $\alpha = 0.3$, $\gamma = 1$, $\delta = 1$.

Panels (c)-(d): folded-node/Hopf bursting example

Equations:

$$
\begin{aligned}
x' &= (y - x^3 + 3x^2 + az)/c \\
y' &= 1 - 5x^2 - y \\
z' &= \varepsilon(\alpha x + \gamma\beta - \delta z) \\
\beta' &= \varepsilon\big(\mu - \gamma_y(y - y_f)^2 - \gamma_\beta(\beta - \beta_f)^2\big)
\end{aligned}
$$

Parameter values:

$a = 1$, $c = 2$, $\varepsilon = 0.004$, $\alpha = 0.3$, $\gamma = 1$, $\delta = 1$, $\mu = 0.0104$, $\gamma_y = 0.0003$ and $\gamma_\beta = -0.05$.

Equations and parameter values for Fig 10.

Panels (a)-(b): fold - fold-of-cycles bursting example

Equations:

$$\begin{aligned} x' &= (y - f(x) + az)/c \\ y' &= -x^3 + A_1(z)x + A_2(z) - y(A_3(z) - x + x^2) \\ z' &= \varepsilon(\alpha x + \gamma\beta - \delta z), \end{aligned}$$

with: $f(x) = 0$, $A_1(z) = 0.1201z + 0.1871$, $A_2(z) = 0.0906z - 0.0251$, $A_3(z) = 0.105z - 0.3526$.

Parameter values:

$a = 0$, $c = 1$, $\varepsilon = 0.01$, $\alpha = 0$, $\gamma = -1$, $\delta = 1$.

Panels (c)-(d): folded-node/fold-of-cycles bursting example

Equations:

$$\begin{aligned} x' &= (y - f(x) + az)/c \\ y' &= -x^3 + A_1(z)x + A_2(z) - y(A_3(z) - x + x^2) \\ z' &= \varepsilon(\alpha x + \gamma\beta - \delta z) \\ \beta' &= \varepsilon\big(\mu - \gamma_y(y - y_f)^2 - \gamma_\beta(\beta - \beta_f)^2\big), \end{aligned}$$

with: $f(x) = 0$, $A_1(z) = 0.1201z + 0.1871$, $A_2(z) = 0.0906z - 0.0251$, $A_3(z) = 0.105z - 0.3526$.

Parameter values:

$a = 0$, $c = 1$, $\varepsilon = 0.01$, $\alpha = 0$, $\gamma = -1$, $\delta = 1$, $\mu = -0.00012$, $\gamma_y = -0.003$, $\gamma_\beta = 0.0001$.

Equations and parameter values for Fig 11.

Equations:

$$\begin{aligned} r' &= 2rf(r) \\ \theta' &= 1 \\ a' &= \varepsilon(r - \beta) \\ \beta' &= \delta\left(\mu - \gamma_r(r - r_f)^2 - \gamma_\beta(\beta - \beta_f)^2\right) \end{aligned}$$

with: $f(r) = 0.01 - (r - 0.5)^4 + 0.25((r - 0.6)^2 - a^2)$.

Parameter values:

(a) $\varepsilon = 10^{-4}$, $\delta = 5.0 \cdot 10^{-4}$, $\mu = -5.0 \cdot 10^{-5}$, $\gamma_r = -0.01$, $r_f = 0.1063$, $\gamma_\beta = -0.01$, $\beta_f = 0.10395$
(b) $\varepsilon = 10^{-4}$, $\delta = 3.5 \cdot 10^{-4}$, $\mu = 5.2 \cdot 10^{-4}$, $\gamma_r = 0.01$, $r_f = 0.6106$, $\gamma_\beta = 0.20$, $\beta_f = 0.611065$
(c) $\varepsilon = -10^{-4}$, $\delta = 1.0 \cdot 10^{-4}$, $\mu = -4.7 \cdot 10^{-4}$, $\gamma_r = -0.10$, $r_f = 0.7848$, $\gamma_\beta = -0.10$, $\beta_f = 0.784525$.

Equations and parameter values for Fig 12.

Equations:

$$
\begin{aligned}
x' &= (2-z)f(x) + f(y) - f(x)r^2(k - r^2)\\
y' &= -f(x) + (2-z)f(y) - f(x)r^2(k - r^2)\\
z' &= \varepsilon(z - g(r))\\
\beta' &= \delta(\mu - \gamma_y(y - y_f)^2 - \gamma_\beta(\beta - \beta_f)^2),
\end{aligned}
$$

with: $f(p) = p\left(z - 1.25(p+4)^2\right)$, $r^2 = x^2 + y^2$, $g(r) = \left(\left(\frac{r-\beta}{0.725}\right)^3/3 - \frac{r-\beta}{0.725}\right)1.195 + 0.78$.

Parameter values:

$k = 3.8$, $\varepsilon = 0.02$, $\delta = -0.002$, $\mu = 0.008$, $\gamma_y = 0.02$, $\gamma_\beta = 0.15$, $y_f = 0$, $\beta_f = 3.175$.

Equations and parameter values for Fig 13.

Equations:

$$
\begin{aligned}
C_m V' &= I - (I_{Ca} + I_{Kdr} + I_{leak} + I_{K1} + I_{K2})\\
n' &= (n_\infty(V) - n)/\tau_n(V)\\
s_1' &= (s_{1\infty}(V) - s_1)/\tau_{s_1}\\
s_2' &= (s_{2\infty}(V) - s_2)/\tau_{s_2}
\end{aligned}
$$

The ionic current are given by

$$
\begin{aligned}
I_{\mathrm{Ca}} &= g_{\mathrm{Ca}} m_\infty(V)\,(V - V_{\mathrm{Ca}})\\
I_{Kdr} &= g_{Kdr} n\,(V - V_K)\\
I_{leak} &= g_{\mathrm{leak}}\,(V - V_{\mathrm{leak}})\\
I_{K1} &= g_{K1} s_1\,(V - V_K)\\
I_{K2} &= g_{K2} s_2\,(V - V_K)
\end{aligned}
$$

with: $x_\infty(V) = \frac{1}{1+\exp((v_x - V)/s_x)}$, $x \in \{m, n, s_1, s_2\}$, and: $\tau_n(V) = \frac{\bar{\tau}_n}{1+\exp((V - v_n)/s_n)}$.

Parameter values (units):

(a1)-(a2): $C_m = 4525$ (fF), $g_{\mathrm{Ca}} = 280$ (pS), $g_{Kdr} = 1300$ (pS), $g_{\mathrm{leak}} = 25$ (pS), $g_{K_1} = 22$ (pS), $g_{K_2} = 16$ (pS), $V_{\mathrm{Ca}} = 100$ (mV), $V_K = -80$ (mV), $V_{leak} = -40$ (mV), $v_m = -22$ (mV), $s_m = 7.5$ (mV), $v_n = -9$ (mV), $s_n = 10$ (mV), $\bar{\tau}_n = 8.25$ (ms), $v_{s_1} = -50$ (mV), $s_{s_1} = 5$ (mV), $\tau_{s_1} = 1000$ (ms), $v_{s_2} = -40$ (mV), $s_{s_2} = 15$ (mV), $\tau_{s_2} = 30000$ (ms).

(b1)-(b2): (same as before except) $g_{K_1} = 18.5$ (pS), $g_{K_2} = 20$ (pS), $v_{s_1} = -51$ (mV), $\tau_{s_1} = 600$ (ms), $v_{s_2} = -35$ (mV), $\tau_{s_2} = 4000$ (ms).
